# Sound-evoked pupil dilation quantifies misophonic symptoms

**DOI:** 10.1371/journal.pone.0348278

**Published:** 2026-05-20

**Authors:** Jan Willem de Gee, Laia Alonso-Marmelstein, Kate Schwarz-Roman, Romke Rouw

**Affiliations:** 1 Swammerdam Institute for Life Sciences, University of Amsterdam, Amsterdam, The Netherlands‌‌; 2 Amsterdam Brain and Cognition, University of Amsterdam, Amsterdam, The Netherlands; 3 Department of Psychology, University of Amsterdam, Amsterdam, The Netherlands; Julius-Maximilians-Universität Würzburg: Julius-Maximilians-Universitat Wurzburg, GERMANY

## Abstract

Misophonia is a debilitating disorder where seemingly innocuous sounds (often human made, such as chewing or throat clearing) evoke intense negative cognitive, emotional and physical “fight-or-flight” responses. Recent studies reported alarmingly high prevalence across different countries and population characteristics, revealing an urgent need for a better understanding of this condition as well as improved measurement and diagnostic tools. First, current misophonia symptom measurements rely on (subjective) self-reports, and different studies employ various diagnostic approaches and cut-off scores. There is an urgent need for a complementary (objective) psychophysiological measurement tool. Second, the role of “mild” or “moderate” symptoms is currently topic of debate: are they still manifestations of the misophonic disorder? Here, we employ pupillometry to map out arousal responses to misophonia trigger sounds. We show that (i) pupil dilation can reliably differentiate misophonic responses from responses to generally unpleasant sounds (such as nails on chalk board), (ii) the “milder cases” of misophonia still show arousal responses characteristic of misophonia, and (iii) pupillometry can even be used to aid diagnosis in an individual; based on only pupil dilation, misophonic symptom severity could be reliably predicted at the level of a single individual. We conclude that even mild misophonic responses can reliably, objectively and cost-effectively be indexed by pupil-linked arousal.

## Introduction

Individuals with misophonia experience strong negative emotions like rage or disgust in response to everyday, often human-made, sounds (e.g., chewing or throat clearing) [1–6]. The ubiquitous nature of the “trigger” stimuli and strength of the misophonic response can lead to severe distress and even suicide ideation [7–9]. Misophonia is a newly defined disorder, quickly gaining scientific attention across scientific disciplines (e.g., audiology, cognitive science, clinical psychology, occupational therapy, psychiatry, and neuroscience) [3–5,7–15]. Despite rapidly advancing research, much remains unknown about the etiology, course, or mechanisms of the condition, leaving open the question why seemingly innocuous daily sounds would evoke such strong aversive (“fight or flight”) responses.‌‌

The need for a better understanding of misophonia extends beyond fundamental scientific interest. A recent series of studies revealed alarmingly high misophonia prevalence numbers of ~1 in 5 in general (non-clinical) population samples across different nations (Turkey [16], UK [17], India [18], China [19], USA [20]). Results furthermore showed a wide range of symptom severity: “severe misophonia” was reported in 5% to 13% of the population [16,20–22] while the same studies also report “some” level of misophonic symptoms in as much as 79% of the population [16,20]. This reflects the current struggle on how to interpret “mild” or “moderate” symptoms: are they still manifestations of a misophonic response? Put differently, can “mild” misophonic responses be discerned from normal annoyance to unpleasant sounds? The call for improved misophonia diagnostic and measurement tools thus entails a need for methodology that reliably distinguishes misophonic from non-misophonic complaints, while simultaneously being sensitive to variations in misophonic response severity.

Misophonia research would benefit from a physiological tool to objectively characterize individual variation in misophonia symptom severity. To date, researchers have largely measured misophonic complaints using self-reports. As the field is quickly expanding, assessment approaches include questionnaires, structured diagnostic interviews and standardized experimental designs [7,23–25]. Yet, misophonia researchers have pointed at the limitations of employing only self-report measurements [23,25], which are prone to response bias: participants may underreport or exaggerate symptoms due to social desirability, memory errors, mood, or personal interpretations. Furthermore, while diagnostic psychiatric criteria have been formulated [3], different studies still employ various diagnostic approaches and different cut-off scores [6,7,10,24], complicating cross-study comparisons or conclusions.

Pupillometry is easy to use, cost-effective, and may be employed on a large scale to objectively and sensitively quantify misophonia severity through sound-evoked pupil responses: (i) trigger sounds elicit exaggerated responses in the anterior insula and anterior cingulate cortex [4,11], key regions of the cortical salience network, (ii) these structures send top-down projections to the subcortical ascending arousal network, including the noradrenergic locus coeruleus [26–28], (iii) ascending arousal nuclei, in turn, increase sympathetic activity (fight-or-flight) and suppresses parasympathetic activity (rest-and-digest) during stress, arousal, and attention [29,30], (v) pupil size fluctuations at constant luminance reflect activity of the cortical saliency network, neuromodulatory activity and autonomic arousal [27,28,31–35], and, as the above circuit would predict, (vi) pupil responses scale with the emotional intensity of auditory stimuli [36–38]. Other physiological measures, such as galvanic skin response and heart rate variability [1,4,11,39], suffer from practical issues in the context of misophonia: these methods are relatively noisy and sluggish [40] and require “on-the-body” measurements (e.g., fitting equipment or on-skin glue), which is a significant drawback for clinical groups. Additionally, neuroimaging [4,11] is expensive and therefore unsuitable for large-scale diagnosis.

How pupillary response correlates with individual misophonic severity has not yet been studied. We hypothesized that the pupil would dilate in response to both generally unpleasant sounds and common misophonia trigger sounds, and, across individuals, the pupil response contrast (trigger vs generally unpleasant) would scale with misophonia severity across its entire spectrum.

## Materials and methods

### Participants

Thirty-five healthy participants (28 females; 18–52 y [median, 23 y]) participated in Experiment 1. Forty-four healthy participants (6 females; 18–64 y [median, 24 y]) participated in Experiment 2. All participants had normal or corrected-to-normal hearing and vision. Participants gave written informed consent and were remunerated by the hour or received credit points. Participants were recruited through University of Amsterdam student recruitment, (online) misophonia support groups, Dr. Rouw’s misophonia research contacts, and word-of-mouth. The experiments were approved by the ethics committee of the Department of Psychology at the University of Amsterdam. Experiment 1 was approved on March 22^nd^, 2023 (FMG-2306) and ran from May 8^th^, 2023, until July 25^th^, 2023. Experiment 2 was approved on February 6^th^, 2025 (FMG-2306; amendment) and ran from February 20^th^, 2025, until May 16^th^, 2025. Five participants from Experiment 1 were excluded due to technical issues. Data from both experiments were pooled, unless stated otherwise.

### Procedure

**Fig 1:**
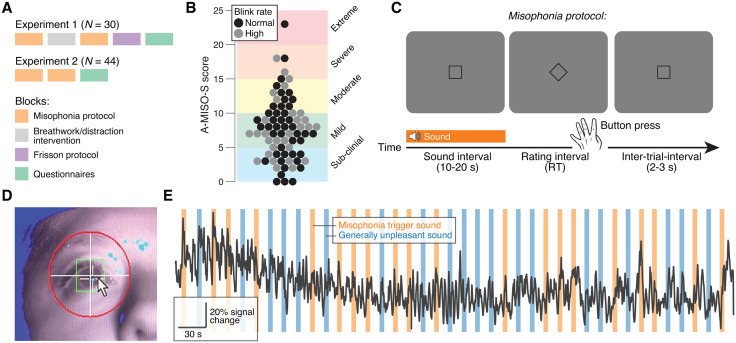
Study design. **(A)** Experimental blocks in experiments 1 and 2 (Materials and Methods). **(B)** Amsterdam Misophonia Scale (A-MISO-S) scores [3]. Every data point is a participant (N = 74); grey data points indicate individuals who blinked on more than two-thirds of sound presentations (Materials and Methods). **(C)** Sequence of sound and rating interval during the misophonia protocol. **(D)** Image of pupil size recordings. **(E)** Example session. Black trace, pupil size time series; colored bars, sounds.

Experiment 1 lasted for 1.5 hours and consisted of the following parts (explained in detail below) (Fig 1A): (i) misophonia protocol (block 1), (ii) breathwork/distraction intervention, (iii) misophonia protocol (block 2), (iv) frisson protocol (two blocks), and (v) post-experiment questionnaires. We collected eye and heart rate data during all sound presentations (steps i, iii and iv). Experiment 2 lasted for 1 hour and was identical to Experiment 1 except the breathwork intervention and frisson blocks were omitted. All acquired data is presented here, except for frisson data.

#### Misophonia protocol.

All participants were asked to sit in a dimly lit room and to rest their head on a chin rest (placed at 50 cm distance in front of the screen) and to maintain central fixation. Each trial consisted of a sound interval, a rating interval and an inter-trial interval. The sound interval showed a blank grey screen and a fixation square, and a sound was presented (duration, 10–20 s; loudness, 30–70 dB). In the rating interval, the fixation square rotated 45° (into a diamond) and participants were prompted to rate how aversive the sound was, on a scale from 1 to 4, with a left-handed button press on the keyboard in front of them: G, “This sound doesn’t affect me at all, the sound may be mildly annoying/disturbing”; H, “This sound is annoying, I don’t like the sound”; J, “I do not like this sound at all, it makes me feel very uncomfortable”; Space bar, “I feel angry, I feel anxiety, I feel fear, I need to flee, I want to remove the headphones, this sound makes me physically upset”. Participants received instructions about this mapping prior to starting the experiment. To avoid ambiguity the scale is ordered to reflect the increasing negative valence/increasing arousal characteristic to misophonia, from neutral to full misophonic response. The G-to-spacebar key arrangement was selected to support a comfortable hand posture and facilitate fast and intuitive response mapping. The trial ended with an inter-trial interval (2–3 s, uniformly distributed). Visual stimuli were displayed on a gamma-corrected monitor (spatial resolution of 2560 by 1440 pixels) with a vertical refresh rate of 100 Hz, and sound stimuli were presented using IMG Stageline MD-5000DR headphones.

In one block, participants were exposed to, and evaluated, ten generally unpleasant sounds, and ten common misophonia trigger sounds, which were each repeated once; the resulting forty sounds were presented in fully randomized order (Fig 1E). We used the following generally unpleasant sounds: baby crying, bicycle breaks, clarinet squeaks, construction drilling, dog barking, fire alarm, glass on metal, modem dialup, nails on a chalkboard, and Styrofoam squeak. We used the following misophonia trigger sounds: apple eating, chip bag crinkling and eating (Cheetos), chip eating, clearing throat, dog eating, gum chewing, keyboard typing, pen clicking, runny nose, and slurping. All sounds were acquired copyright free from https://freesound.org/, except for the clarinet squeak which was recorded by K. Schwarz (one of the authors). We did not match sounds for all acoustic features, as this would distort sounds to the extent that they are no longer recognizable. For example, misophonia triggers are often soft human-made sounds (sniffing, swallowing and whispering), and loudness is an integral part of the sound stimulus. Therefore, we opted for using the same sounds as in previous work [1,41] and, for transparency, plotting the associated spectrograms (S1A Fig) and making the sound stimuli publicly available.

Generally unpleasant sounds occupy specific regions of acoustic space [41,42] (e.g., characteristic frequencies and amplitude modulations) that reliably evoke strong negative autonomic responses, making them robust control stimuli for the misophonic response. In contrast, misophonic trigger sounds are not defined by these “unpleasant” acoustic signatures: e.g., loudness is an aversive psychoacoustic property, but misophonic triggers are commonly soft [1–4,10]. Instead, the misophonic reaction critically depends on more complex cognitive, social, and neurological mechanisms [2,9,10,43], evoking a characteristic fast and high-intensity autonomic misophonic reaction that can feel almost uncontrollable. Consequently, the standard approach of contrasting these two sound categories [1,4,12,44–46] allows to measure the misophonic status of an individual’s physiological and behavioral response to misophonic triggers over “normal” unpleasant sound responses (e.g., it controls for potential individual differences in hearing ability and general sound sensitivity).

#### Breathwork/distraction intervention.

In Experiment 1, participants were randomly allocated into one of two experimental conditions: breathwork or distraction. Each intervention lasted 30 minutes. The breathwork intervention was scripted and recorded by L. Alonso-Marmelstein (a certified breath coach and registered yoga teacher) and included multiple breathing techniques used in previous studies [47]. The specific breathing techniques were belly breathing, 4:4 breathing, 4:4:4:4 box breathing and 4:7:8 breathing. Each technique was presented for an average of 7.5 minutes. Participants were allowed to sit on a chair, cushion or lay down on a yoga mat for the duration of the intervention. We played the training video on a laptop that they could appropriately position. The distraction intervention consisted of continuously playing the game “Snake” from Google.com. The participant used the arrow keys to move a digital snake around the board, and the objective was to not crash into one’s own tail, which kept growing. They were encouraged to beat the highest score set by other participants. The breathwork interventions did not affect any of the reported pupillometry results (S2L,M Fig).

#### Frisson protocol.

In Experiment 1, participants were asked to provide a song that gave them chills, tingles down the spine, welling in the chest, or provided strong physiological sensations during listening. To control for effects of length, loudness, and musical features, the second song was the previous participant’s chosen song [48]. Participants were asked to report the start (z button) and end (m button) of any physical feeling while listening. Frisson data were collected after the experimental conditions reported in this manuscript and do not relate to current results. Frisson data will be the focus of another report.

#### Post-experiment questionnaires.

Participants filled out the Body Consciousness Scale [49] to measure awareness and monitoring of bodily processes, the Amsterdam Misophonia Scale [3] to measure misophonia severity, the STAI-S questionnaire to measure current state of mood [50] and a single question on the autonomous sensory meridian response [51]. While misophonia remains a topic of research, there is evidence that the current understanding and characterization of the condition can be reliably captured with the Amsterdam Misophonia Scale questionnaire [3,52,53] and that the scale is sensitive to variations in the severity of misophonic complaints, such as misophonic symptom reduction following treatment [54–56].

### Eye data acquisition

Pupil size and gaze data were obtained with Eyelink 1000 devices (SR Research, Osgoode, Ontario, Canada) at 1000 Hz with an average spatial resolution of 15–30 min arc (Fig 1D,E).

### Heart rate acquisition

Heart rate and heart rate variability were measured with an electrocardiogram (ECG) device. Three electrodes were placed on the participant’s body: one on either side of their chest, and one on the lower left torso. Heart rate data will be the focus of another report.

### Exclusion criteria

We excluded trials in which participants blinked during the first three seconds of sound presentation and excluded participants for which more than two-thirds of trials had to be excluded due to blinking (N = 30, grey data points in Fig 1B and S1B,C Fig). The relatively high number of excluded participants may be explained by us not explicitly instructing them to refrain from blinking during the sounds. The average blink rate during the first three seconds of sound presentation was 19.5 blinks per minute (S1C Fig), which is close to the average human spontaneous blink rate of ~17 blinks per minute [57,58]. Results based on all data (without having excluded participants and trials) are presented in S2D,E Fig and warrant the same conclusions.

### Analysis of eye data

All analyses were performed using custom-made Python software.

#### Blink and saccade detection.

Periods of blinks and saccades were detected using the manufacturer’s standard algorithms with default settings. We counted the number of blinks that occurred during the first three seconds of each sound presentation.

#### Preprocessing.

We applied to each pupil recording (i) linear interpolation of values measured just before and after each identified blink (interpolation time window, from 200 ms before until 200 ms after blink), (ii) band-pass filtering (third-order Butterworth, passband: 0.01–10 Hz), (iii) removal of pupil responses to blinks and to saccades, by first estimating these responses by means of deconvolution and then removing them from the pupil time series by means of multiple linear regression [59], and (iv) conversion to units of modulation (percent signal change) around the mean of the pupil time series from each block.

#### Quantification of sound-evoked pupil responses.

We computed task-evoked pupil response measures for each trial as the mean of the pupil diameter modulation values in the window 1 s to 3 s from sound onset, minus the mean pupil size during the 0.25 s before sound onset. This time window is based on the known pupil impulse response function [60] and is the same as in recent pupillometry during sound presentation [61,62]. We additionally removed (via linear regression) trial-to-trial variation in the pupil response amplitude that was related to the pre-trial (pre-sound) baseline pupil size (e.g., through reversion to the mean [63]).

### Statistical comparisons

We used a paired samples t-test to quantify the difference in pupil response or subjective rating between trigger and generally unpleasant sounds. We used a mixed linear regression model to quantify the dependence of pupil response magnitude on aversiveness rating (values 1–4) and sound category (0, unpleasant; 1, trigger). The fixed effects were specified as:


$$\begin{array}{c} P\;\sim \;\beta_{0}1+\beta_{1}R+\beta_{2}C \end{array}$$

with P as trial-wise pupil response magnitudes, R as the sound-wise aversiveness ratings, C as sound-wise sound category and β as the regression coefficients. We included the maximal random effects structure justified by the design [64], which meant that intercepts and rating and category coefficients could vary with participant. We quantified across-participant correlations using Pearson’s correlation coefficient.

## Results

### Misophonia severity is reliably predicted by trigger-evoked pupil dilation

We characterized misophonia severity in our sample of seventy-four participants (combined across experiments 1 and 2; Fig 1A) using the *Amsterdam Misophonia Scale* (A-MISO-S) [3] (Materials and Methods). In line with earlier work [9,16,20], we observed substantial variation in a sample drawn from the general population (Fig 1B): 27% was sub-clinical (scores 1–4), 43% experienced mild misophonia (scores 5–9), 21% experienced moderate misophonia (scores 10–14), 7% experienced severe misophonia (scores 15–19) and 1% experienced extreme misophonia (scores 20–24). The same seventy-four participants listened to a sequence of sounds and rated their aversiveness on a four-point scale (Fig 1C; Materials and Methods): half were generally unpleasant (e.g., nails on chalkboard) and half were common misophonia triggers (e.g., clearing throat). See Materials and Methods for full list of sounds and S1A Fig for their spectrograms. We simultaneously recorded pupil size (Fig 1D,E).

**Fig 2:**
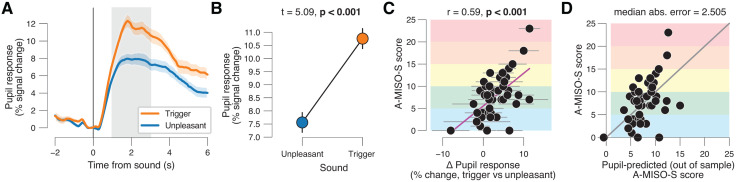
Sound-evoked pupil dilation reliably predicts individual misophonia severity. **(A)** Pupil response time courses separately for sound category (misophonia trigger and generally unpleasant; collapsed across rating), time-locked to sound onset. Shading, S.E.M. across participants (N = 44); grey shading, interval used for quantifying task-evoked pupil responses (Materials and Methods) **(B)** Sound-evoked pupil response magnitude, separately sound category. Error bars, S.E.M. across participants (N = 44); stats, paired samples t-test. **(C)** Individual A-MISO-S score plotted against individual pupil response contrast (trigger vs unpleasant sounds). Each data point is a participant. Error bars, standard deviation of bootstrapped distribution, corresponding to S.E.M; stats, Pearson correlation. **(D)** A-MISO-S score plotted against pupil-predicted A-MISO-S score (leave-one-out cross-validation; Materials and Methods). Each data point is a participant. Solid line, identity line.

We observed robust pupil dilation in response to both misophonia trigger and generally unpleasant sounds (Fig 2A). On average, sound-evoked pupil dilation was larger for misophonia trigger versus generally unpleasant sounds (Fig 2A,B). Given the more subtle acoustic features of misophonia trigger sounds (S1A Fig; Materials and Methods), this group-level effect may seem unexpected at first glance. However, this effect may be explained by the substantial number of participants in our sample with mild to severe misophonia (Fig 1B; average A-MISO-S score of our sample is 8 out of 25).

We next tested if, across individuals, these sound-evoked pupil responses were related to misophonia severity. In line with earlier work [1,4,12,44–46], we controlled for potential confounding individual differences such as hearing ability or general sound sensitivity by computing a pupil response contrast (Materials and Methods): misophonia trigger vs generally unpleasant. This contrast is a standard method in misophonia research, as it specifically reflects the effect of the misophonic trigger, rather than more general emotional, auditory or physiological (pupil) effects. As a first step, we assessed test–retest reliability of pupil responses separately for triggers and generally unpleasant sounds, and for the contrast between them. Reliability was high for trigger sounds (r = 0.805) and generally unpleasant sounds (r = 0.758) but lower for the contrast (r = 0.570) (S2A Fig). This reduction in reliability is consistent with the well-known tendency for difference scores to accumulate measurement error from both component conditions. Next, confirming our hypothesis, we found that the pupil response contrast reliably predicted A-MISO-S scores across participants (Fig 2C). We used leave-one-out cross-validation to quantify the error between pupil-predicted misophonia severity and actual misophonia severity. Specifically, we computed the linear relationship between the A-MISO-S scores and the pupil response contrast (trigger vs unpleasant) based on the data from all participants minus one and then predicted the A-MISO-S score for the left-out participant based on his/her pupil data. This analysis showed that A-MISO-S score could be reliably predicted, based on only pupil dilation, at the level of a single individual (Fig 2D): the median absolute error was 2.505, which is below the granularity of severeness categories of the A-MISO-S.

Several control analyses speak to the robustness of our results. First, we provided an internal replication. In all analyses reported so far, we pooled data from Experiments 1 and 2 (Fig 1A; Materials and Methods), but, critically, A-MISO-S scores and the pupil response contrast are robustly correlated in two independent groups of participants (S2B,C Fig). Second, the result is robust with respect to how eye blinks are treated in the analyses. In all analyses so far, we excluded trials in which participants blinked during the first three seconds of sound presentation (Materials and Methods) and excluded participants for which more than two-thirds of trials had to be excluded due to blinking (N = 30, grey data points in Fig 1B and S1B,C Fig; Materials and Methods), but the results are qualitatively the same when including all participants and trials regardless of blink behavior (S2D,E Fig). Third, the result is robust with respect to pupil data preprocessing choices. We preprocessed the pupil data in the same way as in our recent pupillometry work [65–67] (Materials and Methods), but the results are qualitatively the same when doing less extensive preprocessing (S2F,G Fig). Fourth, using 0–10 seconds as a broader time window of interest to compute sound-evoked pupil responses resulted in an even stronger correlation between A-MISO-S scores the pupil contrast (r = 0.64, p < 0.001). This result may indicate that sound stimuli evoke alertness maintenance over relatively long timescales [68]. Fifth, the result cannot be explained by differences in (micro)-saccade rates (S2H,I Fig).

One possibility is that pupil response variability is smaller for trigger vs generally unpleasant sounds, and that this is especially true for those individuals with severe misophonia. However, we did not find evidence for this: at the group-level there was no difference in pupil response variability between triggers and generally unpleasant sounds (S2J Fig); likewise, there was no significant relationship between A-MISO-S scores and individual pupil response variability contrast (S2K Fig).

In sum, we confirmed our hypothesis that misophonia severity is predicted by evoked pupil dilation to triggers vs generally unpleasant sounds. This suggests that a single individual with unrealistic questionnaire scores (e.g., due to misinterpretation or response bias) might be detectable by adding pupillometry as an objective tool.

### Trigger-evoked pupil dilation and subjective aversiveness ratings each explain unique variance in misophonia severity

**Fig 3:**
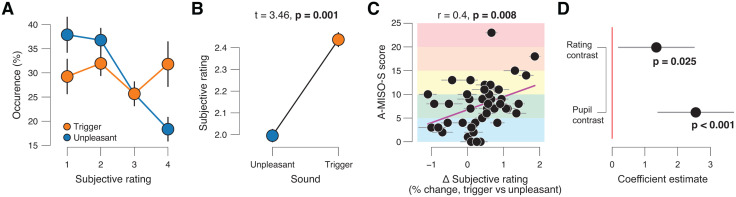
Sound-evoked pupil dilation reliably predicts individual misophonia severity. **(A)** Subjective rating occurrence, separately for misophonic triggers and generally unpleasant sounds. A higher number reflects a more aversive subjective rating (Materials and Methods). Error bars, S.E.M. across participants (N = 44). **(B)** Mean subjective rating, separately for misophonic triggers and generally unpleasant sounds. Error bars, S.E.M. across participants (N = 44); stats, paired samples t-test. **(C)** Individual A-MISO-S score plotted against individual subjective rating contrast (trigger vs unpleasant sounds). Each data point is a participant. Error bars, standard deviation of bootstrapped distribution, corresponding to S.E.M; stats, Pearson correlation. **(D)** Coefficients of across-subjects multiple regression model in which we regressed A-MISO-S scores on pupil response and subjective rating contrasts (both trigger vs generally unpleasant). Error bars, 95% confidence interval.

After each sound presentation, participants rated its aversiveness on a four-point ordinal scale (Fig 1C), ranging from neutral to full misophonic response (Fig 3A; Materials and Methods). Because of the chosen definitions associated with each rating (Materials and Methods), we assumed that they are ordered with interval properties. This has the benefit that we could repeat the same analyses as we performed for sound-evoked pupil responses.

Three analyses indicated that subjective ratings and evoked pupil responses are related. First, we observed larger pupil responses evoked by sounds that were rated as more aversive (S3A Fig). Second, mimicking the group-level pupil responses (Fig 2A, B), subjective ratings were higher (more negative) for misophonia trigger versus generally unpleasant sounds (Fig 3B). Third, again mimicking the pupil results (Fig 2C), A-MISO-S scores were positively related to the subjective rating contrast (Fig 3C).

However, two results show that aversiveness ratings and pupil responses can also diverge. First, sound-evoked pupil responses were also larger for misophonia trigger sounds when matched for subjective aversiveness rating (S3B Fig; mixed linear model: main effects for aversiveness rating [*z* = 6.790, *p* < 0.001] and sound category [*z* = 3.031, *p* = 0.002]). Thus, for the same subjective aversiveness rating, the pupil dilated more in response to misophonia trigger sounds, than generally unpleasant sounds. In this regression model the average (± S.E.M.; across participants) variance inflation factor (VIF) is 1.27 (± 0.06), which is considerably lower than common cutoffs 5 or 10. Second, the test-retest reliability of subjective ratings was higher than that of pupil responses (trigger sounds, r = 0.942; generally unpleasant sounds, r = 0.920; contrast, r = 0.899) (S3C Fig). Third, we observed that the pupil response contrast (trigger vs unpleasant) was not significantly correlated to the aversiveness rating contrast (trigger vs unpleasant) across participants (S3D Fig). In other words, those participants who rated the misophonia trigger sounds as more aversive than the generally unpleasant sounds did not necessarily also have larger pupil responses during trigger versus unpleasant sounds. Thus, evoked pupil responses and subjective ratings could, in principle, predict unique parts of the across-subjects variance in misophonia severity, as measured by the A-MISO-S.

We used multiple linear regression to assess the unique contribution of subjective ratings and pupil responses in predicting A-MISO-S scores. In this multiple regression model, both measures significantly predicted misophonia severity (Fig 3D; pupil response contrast, *t* = 4.414, *p* < 0.001; aversiveness ratings contrast, *t* = 2.335, *p* = 0.025). We observed the same qualitative result when excluding one participant with severe misophonia (A-MISO-S score > 20) from the analysis, who could be considered an outlier in our sample (pupil response contrast, *t* = 3.580, *p* = 0.001; aversiveness rating contrast, *t* = 2.542, *p* = 0.015).

In sum, sound-evoked pupil dilation and subjective aversiveness ratings each explain unique variance in misophonia severity. This highlights the importance of adding an objective physiological measurement to misophonia diagnosis.

## Discussion

Our study demonstrates for the first time that pupillometry is a sensitive method for measuring variations in misophonic responses, even in milder cases. Pupil response discerned misophonic responses to “trigger” sounds from normal aversive responses to generally unpleasant sounds. Furthermore, pupillometry proved sensitive to variations in the intensity of the misophonic response across different individuals and may therefore be used to aid diagnosis of the condition.

Our finding that pupillometry can reliably measure (variations in) misophonic severity suggests it is a valuable addition to self-report paradigms. While self-report is an important tool to measure phenomenological and emotional experiences, it has some intrinsic weaknesses including unintentional (e.g., misunderstandings or biases) and intentional (malingering) measurement errors. Furthermore, our results show how the (continuous) pupillometry variance can explain additional variance over the (categorical) ratings for misophonic sounds. Thus, adding pupillometry to the misophonia diagnostic toolbox could greatly help deciphering out the different types and severities within a group of individuals reporting misophonic complaints.

Previous studies measured increased autonomic nervous system response in galvanic skin response and heart rate variability [1,4,11,39] and shown how the involuntary emotional and physiological response is an essential characteristic of misophonia [10]. In these previous studies, only the autonomic nervous system response to misophonic sounds in misophonic versus non-misophonic participants was obtained. Our current goal was to add an objective measurement that would provide a more sensitive measurement of across-subject variations in autonomic nervous system response. Pupil size fluctuations at constant luminance have previously been shown to reflect neuromodulatory activity and emotional arousal associated with increased sympathetic activity [28,31–34]. The signal-to-noise ratio of pupil responses is higher compared to galvanic skin heart rate (variability) responses [40]. Furthermore, the temporal resolution of pupillometry is higher compared to functional MRI and physiological measures like the galvanic skin response. Together, this allowed us to take previous findings a step further and predict that pupillometry measurements reflect variations in the strength of misophonic responses (between participants, or between trials). Indeed, our results reflected such response variation, even in moderate and mild cases of misophonia.

There is also a practical reason to add pupillometry to the misophonia toolbox. As compared with neuroimaging or electrophysiological techniques, pupillometry is cheap, mobile, and non-invasive. This is not only relevant to misophonia research, as a similar quest for objective measurements exist in the pluriform and extensive research on sound sensitivity in related research areas (e.g., autism, tinnitus, hyperacusis, or PTSD [69–71]. A recent preprint showed that emotionally evocative sounds elicited abnormally large pupil dilations in a “disordered hearing” (tinnitus and hyperacusis) group [72]. This invites further research of employing pupillometry in sound sensitivity in other conditions. We propose that pupillometry could be employed as a non-invasive, relatively easy-to-use and cost-effective manner to map out and compare responses both within and across the different conditions. An additional advantage is that this methodology can even be used in cases where self-report is limited (e.g., due to age effects or language impairments of the participant group).

There are, however, several limitations to the use of pupillometry in this context. First, there are known time-scale effects on the pupil size timeseries [68,73,74]. However, since we fully randomized sounds within blocks, time-scale effects could have only added noise and could not have driven the result. Second, the signal-to-noise ratio of pupil responses is limited, as illustrated by the modest test-retest reliability of the pupil contrast (S2A Fig; ICC = 0.497). This level of reliability implies that the observed correlation with misophonia severity is likely an underestimate of the true association, because measurement error in the predictor attenuates observed correlations. Thus, the reliability estimate qualifies the precision of the effect size while supporting, rather than undermining, the robustness of the reported relationship. Third, pupillometry is relatively unspecific. For example, pupil dilation can also reflect positive emotional reactions to sounds in frisson [48] and the autonomous sensory meridian response [51]. We dealt with this by presenting sounds that would only evoke negative responses (Materials and Methods), and by computing the sound-evoked pupil contrast (trigger vs generally unpleasant) [1,4,12,44–46], as it controls for potential individual differences such as hearing ability and general sound sensitivity. At the level of the central nervous system, it has been shown that pupil size fluctuations at constant luminance reflect the activity of the cortical salience network (including the insula) [28] as well as that of a number of subcortical neuromodulatory structures [34], including the noradrenergic locus coeruleus [28,35,75,76], the cholinergic basal forebrain [28,63,75], and likely others [77,78]. Thus, future studies should pinpoint the exact neuroanatomical and neurochemical source(s) of our observed effects. Similarly, the current study did not examine which cognitive, social, or emotional factors are associated with the increased pupil response to misophonic sounds (e.g., disgust [38,79]). Exploring the role of such factors with pupillometry represent important directions for future misophonia research.

There are several further limitations to our study. First, follow-up studies can elucidate whether choices in experimental design may increase or decrease obtained effects. In particular, a different set of misophonic/unpleasant sound stimuli may affect the magnitude of the autonomic nervous system (and therefore pupil) response. For example, we choose to use the same standard stimuli for all participants, but using participants’ individual misophonic trigger sounds may lead to different results. However, we expect that such a change in design would only enhance our current results. Second, differences in low-level acoustic features may have affected our group-level analyses. Matching for all acoustic features is however impossible, as this would distort sounds to the extent that they are no longer recognizable (Materials and Methods). Critically, our main conclusions rely on individual differences analyses; since all participants were exposed to the exact same sounds, these analyses do not suffer from potential systematic differences in low-level acoustic features. Third, analyzing which trigger sound most strongly drives pupil responses in an individual, or a more in-depth analysis of individual differences in misophonia was not feasible in the current design, due to limitations in the number of trials and participants. This remains a topic for future research. Fourth, the subjective ratings were measured on a four-point ordinal scale, while evoked-pupil responses were measured on a continuous scale, which may have favored the pupil predictor in the multiple linear regression model. Future work could measure subjective ratings on a continuous scale. Finally, because of the recruitment method and limited number of participants, our results cannot be used to estimate misophonia prevalence in the general population.

The current results reflect how even milder misophonic cases can experience emotional disturbances related to “triggers” in their normal daily life. There are practical implications to these findings, as for sufferers from misophonia the relatively unknown and ill-understood status of the condition impedes explaining misophonia to others, or even understanding their misophonic responses themselves [1,3,7,9]. Adding pupillometry as an objective misophonia measurement tool can improve this situation. Pupil dilation can clearly display the physical response of the sufferers to the triggers. Such objective validation can bring relief to sufferers and improve understanding from their environment. Furthermore, the current lack of objective diagnostic tools hinders rigorous assessment of the efficacy of different treatment approaches of misophonia [8,10]. Our findings suggest that pupillometry can help assess strategies and evaluate new treatments and thus help build effective treatments for this debilitating condition.

## Supporting information

S1 Fig (A) Spectrograms of all sounds. (B) Blink rate during first three seconds of sound presentation. Data is from all participants and all trials (N = 74; before exclusion; Materials and Methods). (C) Histogram of percentage of trials in which participants blinked during the first three seconds of sound presentation (Materials and Methods). Red dashed line, cut off for participant exclusion (66.7%; Materials and Methods).
(EPS)

S2 Fig (A) Bootstrapped distribution of test-retest reliability of pupil measures. Colored tick marks, median of distribution. We bootstrapped the data 10K times separately per participant (with replacement; until the same data set size) and computed the average participant-wise pupil response evoked by triggers, evoked by generally unpleasant sounds, and their contrast. We then correlated pupil measures of neighboring bootstraps across participants and build a bootstrapped distribution of correlation coefficients. Additionally, we computed the intra-class correlation coefficient [80] of the pupil contrast between odd and even sound presentations, based on a mean-rating (k = 2), absolute-agreement, 2-way mixed-effects model: ICC = 0.497, F(43,43)=1.920, p = 0.018, CI95%=[0.05,0.72]. (B,C) As main Fig 2C, but separately for Experiments 1 and 2 (Materials and Methods). (D,E) As main Fig 2A,C but for all data from all participants and all trials (N = 74; before exclusion; Materials and Methods). (F,G) As main Fig 2A,C but after less extensive preprocessing of pupil data: only blink interpolation and conversion to percent signal change. (H,I) As main Fig 2B,C but for saccade rate during the first three seconds of sounds presentation. (J,K) As main Fig 2B,C but for pupil response variability (s.d.). (L) Pupil response (collapsed across sound category) in Experiment 1, separately per experimental manipulation (breathwork [N = 14] vs distraction [N = 8]) and block number. Breathwork reduced the magnitude of sound-evoked pupil responses (irrespective of sound category) in block 2 vs block 1 (p = 0.006). Distraction had no such effect (p = 0.621). The breathwork-related change in pupil response magnitude was not larger than the distraction-related change (p = 0.821). (M) Pupil response contrast (trigger vs unpleasant sounds) in Experiment 1, separately per experimental manipulation (distraction versus breathwork) and block number. There was no effect of breathwork (p = 0.096) or distraction (p = 0.687) on pupil response contrast in block 2 vs block 1, and no difference in this effect between conditions (p = 0.353).
(EPS)

S3 Fig (A) Pupil response time courses separately for subjective rating (of physical and emotional discomfort on a 4-point scale; collapsed across sound category), time-locked to sound onset. Grey shading, interval used for quantifying task-evoked pupil responses (Materials and Methods). (B) Sound-evoked pupil response magnitude, separately per rating and sound category. (C) Bootstrapped distribution of test-retest reliability of rating contrast (Methods). Colored tick marks, median of distribution. Additionally, we computed the intra-class correlation coefficient [80] of the pupil contrast between odd and even sound presentations, based on a mean-rating (k = 2), absolute-agreement, 2-way mixed-effects model: ICC = 0.869, F(43,43)=1.920, p < 0.001, CI95%=[0.76,0.93]. (D) Individual pupil response contrast (trigger vs unpleasant sounds) plotted against individual subjective rating contrast (trigger vs unpleasant sounds). Each data point is a participant. Error bars, standard deviation of bootstrapped distribution, corresponding to S.E.M. Stats, Pearson correlation.
(EPS)

## References

[1] Edelstein M, Brang D, Rouw R, Ramachandran VS. Misophonia: Physiological investigations and case descriptions. Front Hum Neurosci. 2013;7:296. doi: 10.3389/fnhum.2013.00296 23805089 PMC3691507

[2] Jastreboff PJ, Jastreboff MM. The neurophysiological approach to misophonia: Theory and treatment. Front Neurosci. 2023;17:895574. doi: 10.3389/fnins.2023.895574 37034168 PMC10076672

[3] Schröder A, Vulink N, Denys D. Misophonia: Diagnostic criteria for a new psychiatric disorder. PLoS One. 2013;8(1):e54706. doi: 10.1371/journal.pone.0054706 23372758 PMC3553052

[4] Kumar S, Tansley-Hancock O, Sedley W, Winston JS, Callaghan MF, Allen M, et al. The brain basis for misophonia. Curr Biol. 2017;27(4):527–33. doi: 10.1016/j.cub.2016.12.048 28162895 PMC5321671

[5] Ash PA, Benzaquén E, Gander PE, Berger JI, Kumar S. Mimicry in misophonia: A large-scale survey of prevalence and relationship with trigger sounds. J Clin Psychol. 2024;80(1):186–97. doi: 10.1002/jclp.23605 37850971

[6] Andermane N, Bauer M, Sohoglu E, Simner J, Ward J. A phenomenological cartography of misophonia and other forms of sound intolerance. iScience. 2023;26(4):106299. doi: 10.1016/j.isci.2023.106299 37153450 PMC10156615

[7] Jager I, de Koning P, Bost T, Denys D, Vulink N. Misophonia: Phenomenology, comorbidity and demographics in a large sample. PLoS One. 2020;15(4):e0231390. doi: 10.1371/journal.pone.0231390 32294104 PMC7159231

[8] Paunovic KŽ, Milenković SM. The proposed criteria for high perceived misophonia in young healthy adults and the association between Misophonia symptoms and noise sensitivity. Noise Health. 2022;24(113):40–8. doi: 10.4103/nah.nah_40_20 35900389 PMC9703820

[9] Rouw R, Erfanian M. A large-scale study of misophonia. J Clin Psychol. 2018;74(3):453–79. doi: 10.1002/jclp.22500 28561277

[10] Swedo SE, Baguley DM, Denys D, Dixon LJ, Erfanian M, Fioretti A, et al. Consensus definition of misophonia: A delphi study. Front Neurosci. 2022;16:841816. doi: 10.3389/fnins.2022.841816 35368272 PMC8969743

[11] Schröder A, van Wingen G, Eijsker N, San Giorgi R, Vulink NC, Turbyne C, et al. Misophonia is associated with altered brain activity in the auditory cortex and salience network. Sci Rep. 2019;9(1):7542. doi: 10.1038/s41598-019-44084-8 31101901 PMC6525165

[12] Siepsiak M, Rosenthal MZ, Raj-Koziak D, Dragan W. Psychiatric and audiologic features of misophonia: Use of a clinical control group with auditory over-responsivity. J Psychosom Res. 2022;156:110777. doi: 10.1016/j.jpsychores.2022.110777 35259551

[13] Rinaldi LJ, Simner J. Mental health difficulties in children who develop misophonia: An examination of ADHD, depression & anxiety. Child Psychiatry Hum Dev. 2023. doi: 10.1007/s10578-023-01569-y PMC11928355 37501042

[14] Hansen HA, Leber AB, Saygin ZM. The effect of misophonia on cognitive and social judgments. PLoS One. 2024;19(5):e0299698. 38722993 10.1371/journal.pone.0299698 PMC11081244

[15] Guetta RE, Siepsiak M, Shan Y, Frazer-Abel E, Rosenthal MZ. Misophonia is related to stress but not directly with traumatic stress. PLoS One. 2024;19(2):e0296218. doi: 10.1371/journal.pone.0296218 38386641 PMC10883556

[16] Kılıç C, Öz G, Avanoğlu KB, Aksoy S. The prevalence and characteristics of misophonia in Ankara, Turkey: Population-based study. BJPsych Open. 2021;7(5):e144. doi: 10.1192/bjo.2021.978 34353403 PMC8358974

[17] Vitoratou S, Hayes C, Uglik-Marucha N, Pearson O, Graham T, Gregory J. Misophonia in the UK: Prevalence and norms from the S-Five in a UK representative sample. PLoS One. 2023;18(3):e0282777. doi: 10.1371/journal.pone.0282777 36947525 PMC10032546

[18] Sujeeth PR, Hanji R, Nayyar K, Prabhu P. Estimation of prevalence of misophonia among high school students in India. Indian J Otolaryngol Head Neck Surg. 2024;76(2):1678–81. doi: 10.1007/s12070-023-04382-0 38566702 PMC10982162

[19] Zhou X, Wu MS, Storch EA. Misophonia symptoms among Chinese university students: Incidence, associated impairment, and clinical correlates. Journal of Obsessive-Compulsive and Related Disorders. 2017;14:7–12. doi: 10.1016/j.jocrd.2017.05.001

[20] Dixon LJ, Schadegg MJ, Clark HL, Sevier CJ, Witcraft SM. Prevalence, phenomenology, and impact of misophonia in a nationally representative sample of U.S. adults. J Psychopathol Clin Sci. 2024;133(5):403–12. doi: 10.1037/abn0000904 38780601

[21] Williams ZJ, Cascio CJ, Woynaroski TG. Psychometric validation of a brief self-report measure of misophonia symptoms and functional impairment: The duke-vanderbilt misophonia screening questionnaire. Front Psychol. 2022;13:897901. doi: 10.3389/fpsyg.2022.897901 35936331 PMC9355318

[22] Jakubovski E, Müller A, Kley H, de Zwaan M, Müller-Vahl K. Prevalence and clinical correlates of misophonia symptoms in the general population of Germany. Front Psychiatry. 2022;13:1012424. doi: 10.3389/fpsyt.2022.1012424 36479555 PMC9720274

[23] Guetta RE, Cassiello-Robbins C, Anand D, Rosenthal MZ. Development and psychometric exploration of a semi-structured clinical interview for Misophonia. Personality and Individual Differences. 2022;187:111416. doi: 10.1016/j.paid.2021.111416

[24] Simner J, Rinaldi LJ, Ward J. An automated online measure for misophonia: The Sussex Misophonia Scale for Adults. Assessment. 2024;31(8):1598–614. doi: 10.1177/10731911241234104 38414185 PMC11528938

[25] Trumbull J, Lanier N, McMahon K, Guetta R, Rosenthal MZ. Using a standardized sound set to help characterize misophonia: The International Affective Digitized Sounds. PLoS One. 2024;19(5):e0301105. doi: 10.1371/journal.pone.0301105 38776286 PMC11111082

[26] Aston-Jones G, Cohen JD. An integrative theory of locus coeruleus-norepinephrine function: Adaptive gain and optimal performance. Annu Rev Neurosci. 2005;28:403–50. doi: 10.1146/annurev.neuro.28.061604.135709 16022602

[27] Joshi S, Gold JI. Context-dependent relationships between locus coeruleus firing patterns and coordinated neural activity in the anterior cingulate cortex. Elife. 2022;11:e63490. doi: 10.7554/eLife.63490 34994344 PMC8765756

[28] de Gee JW, Colizoli O, Kloosterman NA, Knapen T, Nieuwenhuis S, Donner TH. Dynamic modulation of decision biases by brainstem arousal systems. Elife. 2017;6:e23232. doi: 10.7554/eLife.23232 28383284 PMC5409827

[29] Thiele A, Bellgrove MA. Neuromodulation of Attention. Neuron. 2018;97:769–85. doi: 10.1016/j.neuron.2018.01.008 29470969 PMC6204752

[30] Poe GR, Foote S, Eschenko O, Johansen JP, Bouret S, Aston-Jones G, et al. Locus coeruleus: A new look at the blue spot. Nat Rev Neurosci. 2020;21(11):644–59. doi: 10.1038/s41583-020-0360-9 32943779 PMC8991985

[31] Murphy PR, O’Connell RG, O’Sullivan M, Robertson IH, Balsters JH. Pupil diameter covaries with BOLD activity in human locus coeruleus. Hum Brain Mapp. 2014;35(8):4140–54. doi: 10.1002/hbm.22466 24510607 PMC6869043

[32] Lloyd B, de Voogd LD, Mäki-Marttunen V, Nieuwenhuis S. Pupil size reflects activation of subcortical ascending arousal system nuclei during rest. Elife. 2023;12:e84822. doi: 10.7554/eLife.84822 37367220 PMC10299825

[33] Bradley MM, Miccoli L, Escrig MA, Lang PJ. The pupil as a measure of emotional arousal and autonomic activation. Psychophysiology. 2008;45(4):602–7. doi: 10.1111/j.1469-8986.2008.00654.x 18282202 PMC3612940

[34] Joshi S, Gold JI. Pupil size as a window on neural substrates of cognition. Trends Cogn Sci. 2020;24(6):466–80. doi: 10.1016/j.tics.2020.03.005 32331857 PMC7271902

[35] Joshi S, Li Y, Kalwani RM, Gold JI. Relationships between pupil diameter and neuronal activity in the locus coeruleus, colliculi, and cingulate cortex. Neuron. 2016;89:221–34. doi: 10.1016/j.neuron.2015.11.028 26711118 PMC4707070

[36] Zekveld AA, Koelewijn T, Kramer SE. The pupil dilation response to auditory stimuli: Current state of knowledge. Trends Hear. 2018;22:2331216518777174. doi: 10.1177/2331216518777174 30249172 PMC6156203

[37] McCulloch K, Dalmaijer ES, Rieger G, O’Gorman R. Differences in pupil size during self-reported experiences of disgust, sadness, fear, anger, and happiness. Biol Psychol. 2025;198:109044. doi: 10.1016/j.biopsycho.2025.109044 40320036

[38] Oszczapinska U, Park S, Qiu Y, Nance B, Julien M, Heller LM. The impact of disgusting sounds on pupil diameter of misophonic and non-misophonic listeners. Psychophysiology. 2025;62(2):e70014. doi: 10.1111/psyp.70014 39981631 PMC11843529

[39] Grossini E, Stecco A, Gramaglia C, De Zanet D, Cantello R, Gori B, et al. Misophonia: Analysis of the neuroanatomic patterns at the basis of psychiatric symptoms and changes of the orthosympathetic/ parasympathetic balance. Front Neurosci. 2022;16:827998. doi: 10.3389/fnins.2022.827998 36033627 PMC9406292

[40] Tursky B, Shapiro D, Crider A, Kahneman D. Pupillary, heart rate, and skin resistance changes during a mental task. J Exp Psychol. 1969;79(1):164–7. doi: 10.1037/h0026952 5785627

[41] Kumar S, Forster HM, Bailey P, Griffiths TD. Mapping unpleasantness of sounds to their auditory representation. J Acoust Soc Am. 2008;124(6):3810–7. doi: 10.1121/1.3006380 19206807

[42] Halpern DL, Blake R, Hillenbrand J. Psychoacoustics of a chilling sound. Percept Psychophys. 1986;39(2):77–80. doi: 10.3758/bf03211488 3725541

[43] Berger JI, Gander PE, Kumar S. A social cognition perspective on misophonia. Philos Trans R Soc Lond B Biol Sci. 2024;379(1908):20230257. doi: 10.1098/rstb.2023.0257 39005025 PMC11444241

[44] Kumar S, Dheerendra P, Erfanian M, Benzaquén E, Sedley W, Gander PE, et al. The motor basis for misophonia. J Neurosci. 2021;41(26):5762–70. doi: 10.1523/JNEUROSCI.0261-21.2021 34021042 PMC8244967

[45] Savard M-A, Sares AG, Coffey EBJ, Deroche MLD. Specificity of affective responses in misophonia depends on trigger identification. Front Neurosci. 2022;16:879583. doi: 10.3389/fnins.2022.879583 35692416 PMC9179422

[46] Daniels EC, Rodriguez A, Zabelina DL. Severity of misophonia symptoms is associated with worse cognitive control when exposed to misophonia trigger sounds. PLoS One. 2020;15(1):e0227118. doi: 10.1371/journal.pone.0227118 31945068 PMC6964854

[47] Hopper SI, Murray SL, Ferrara LR, Singleton JK. Effectiveness of diaphragmatic breathing for reducing physiological and psychological stress in adults: A quantitative systematic review. JBI Database System Rev Implement Rep. 2019;17(9):1855–76. doi: 10.11124/JBISRIR-2017-003848 31436595

[48] Laeng B, Eidet LM, Sulutvedt U, Panksepp J. Music chills: The eye pupil as a mirror to music’s soul. Conscious Cogn. 2016;44:161–78. doi: 10.1016/j.concog.2016.07.009 27500655

[49] Miller LC, Murphy R, Buss AH. Consciousness of body: Private and public. Journal of Personality and Social Psychology. 1981;41(2):397–406. doi: 10.1037/0022-3514.41.2.397

[50] Spielberger CD, Gonzalez-Reigosa F, Martinez-Urrutia A, Natalicio LFS, Natalicio DS. Revista Interamericana de Psicología/Interamerican Journal of Psychology. 2017;5. 10.30849/rip/ijp.v5i3%20&%204.620

[51] Valtakari NV, Hooge ITC, Benjamins JS, Keizer A. An eye-tracking approach to Autonomous sensory meridian response (ASMR): The physiology and nature of tingles in relation to the pupil. PLoS One. 2019;14(12):e0226692. doi: 10.1371/journal.pone.0226692 31877152 PMC6932793

[52] Naylor J, Caimino C, Scutt P, Hoare DJ, Baguley DM. The Prevalence and Severity of Misophonia in a UK Undergraduate Medical Student Population and Validation of the Amsterdam Misophonia Scale. Psychiatr Q. 2021;92(2):609–19. doi: 10.1007/s11126-020-09825-3 32829440 PMC8110492

[53] Cakiroglu S, Cosgun S, Gormez V. The prevalence and severity of misophonia in the Turkish population and validation of the Amsterdam Misophonia Scale-Revised. Bull Menninger Clin. 2022;86(2):159–80. doi: 10.1521/bumc.2022.86.2.159 35647776

[54] Schneider RL, Arch JJ. Case study: A novel application of mindfulness- and acceptance-based components to treat misophonia. Journal of Contextual Behavioral Science. 2017;6(2):221–5. doi: 10.1016/j.jcbs.2017.04.003

[55] Reid AM, Guzick AG, Gernand A, Olsen B. Intensive cognitive-behavioral therapy for comorbid misophonic and obsessive-compulsive symptoms: A systematic case study. Journal of Obsessive-Compulsive and Related Disorders. 2016;10:1–9. doi: 10.1016/j.jocrd.2016.04.009

[56] Kamody RC, Del Conte GS. Using dialectical behavior therapy to treat misophonia in adolescence. Prim Care Companion CNS Disord. 2017;19(5):17l02105. doi: 10.4088/PCC.17l02105 28922587

[57] Tomashin A, Fusina F, Marino M, Angrilli A. The eye, a spy hole on human mind: Spontaneous blink rate and amplitude, and their variability, as new psychobiological markers of anxiety. PLoS One. 2025;20(12):e0338262. doi: 10.1371/journal.pone.0338262 41370259 PMC12694841

[58] Bentivoglio AR, Bressman SB, Cassetta E, Carretta D, Tonali P, Albanese A. Analysis of blink rate patterns in normal subjects. Mov Disord. 1997;12(6):1028–34. doi: 10.1002/mds.870120629 9399231

[59] Knapen T, de Gee JW, Brascamp J, Nuiten S, Hoppenbrouwers S, Theeuwes J. Cognitive and ocular factors jointly determine pupil responses under equiluminance. PLoS One. 2016;11(5):e0155574. doi: 10.1371/journal.pone.0155574 27191166 PMC4871560

[60] Hoeks B, Levelt WJM. Pupillary dilation as a measure of attention: A quantitative system analysis. Behavior Research Methods, Instruments, & Computers. 1993;25(1):16–26. doi: 10.3758/bf03204445

[61] Becker J, Viertler M, Korn CW, Blank H. The pupil dilation response as an indicator of visual cue uncertainty and auditory outcome surprise. Eur J Neurosci. 2024;59(10):2686–701. doi: 10.1111/ejn.16306 38469976

[62] Doll L, Heiland S, Gutschalk A. A role of pupil-linked arousal, cingulo-insular cortex, and intralaminar thalamus for auditory near-threshold perception. J Cogn Neurosci. 2025;37(9):1391–415. doi: 10.1162/jocn_a_02324 40136289

[63] Mridha Z, de Gee JW, Shi Y, Alkashgari R, Williams J, Suminski A, et al. Graded recruitment of pupil-linked neuromodulation by parametric stimulation of the vagus nerve. Nat Commun. 2021;12(1):1539. doi: 10.1038/s41467-021-21730-2 33750784 PMC7943774

[64] Barr DJ, Levy R, Scheepers C, Tily HJ. Random effects structure for confirmatory hypothesis testing: Keep it maximal. J Mem Lang. 2013;68(3):10.1016/j.jml.2012.11.001. doi: 10.1016/j.jml.2012.11.001 24403724 PMC3881361

[65] Hebisch J, Ghassemieh A-C, Zhecheva E, Brouwer M, van Gaal S, Schwabe L, et al. Task-irrelevant stimuli reliably boost phasic pupil-linked arousal but do not affect decision formation. Sci Rep. 2024;14(1):28380. doi: 10.1038/s41598-024-78791-8 39551856 PMC11570621

[66] de Gee JW, Kloosterman NA, Braun A, Donner TH. Catecholamines reduce choice history biases in perceptual decision making. PLoS Biol. 2025;23(9):e3003361. doi: 10.1371/journal.pbio.3003361 40901785 PMC12407465

[67] de Gee JW, Correa CMC, Weaver M, Donner TH, van Gaal S. Pupil dilation and the slow wave ERP reflect surprise about choice outcome resulting from intrinsic variability in decision confidence. Cereb Cortex. 2021;31(7):3565–78. doi: 10.1093/cercor/bhab032 33822917 PMC8196307

[68] Yamashita J, Terashima H, Yoneya M, Maruya K, Oishi H, Kumada T. Pupil trend reflects suboptimal alertness maintenance over 10 s in vigilance and working memory performance: An exploratory study. eNeuro. 2024;11. doi: 10.1523/ENEURO.0250-24.2024 PMC11656038 39557569

[69] Stiegler LN, Davis R. Understanding sound sensitivity in individuals with autism spectrum disorders. Focus Autism Other Dev Disabl. 2010;25(2):67–75. doi: 10.1177/1088357610364530

[70] Greenberg B, Carlos M. Psychometric properties and factor structure of a new scale to measure hyperacusis: introducing the inventory of hyperacusis symptoms. Ear Hear. 2018;39(5):1025–34. doi: 10.1097/AUD.0000000000000583 29742543

[71] Jüris L, Andersson G, Larsen HC, Ekselius L. Psychiatric comorbidity and personality traits in patients with hyperacusis. Int J Audiol. 2013;52(4):230–5. doi: 10.3109/14992027.2012.743043 23244506

[72] Smith SS, Jahn KN, Sugai JA, Hancock KE, Polley DB. The human pupil and face encode sound affect and provide objective signatures of tinnitus and auditory hypersensitivity disorders. bioRxiv. 2023. doi: 10.1101/2023.12.22.571929

[73] de Gee JW, Mridha Z, Hudson M, Shi Y, Ramsaywak H, Smith S, et al. Strategic stabilization of arousal boosts sustained attention. Curr Biol. 2024;34(18):4114-4128.e6. doi: 10.1016/j.cub.2024.07.070 39151432 PMC11447271

[74] Brink RL van den, Murphy PR, Nieuwenhuis S. Pupil diameter tracks lapses of attention. PLOS ONE. 2016;11:e0165274. doi: 10.1371/journal.pone.0165274 PMC5074493 27768778

[75] Reimer J, McGinley MJ, Liu Y, Rodenkirch C, Wang Q, McCormick DA, et al. Pupil fluctuations track rapid changes in adrenergic and cholinergic activity in cortex. Nat Commun. 2016;7:13289. doi: 10.1038/ncomms13289 27824036 PMC5105162

[76] Breton-Provencher V, Sur M. Active control of arousal by a locus coeruleus GABAergic circuit. Nat Neurosci. 2019;22(2):218–28. doi: 10.1038/s41593-018-0305-z 30643295 PMC6385895

[77] Cazettes F, Reato D, Morais JP, Renart A, Mainen ZF. Phasic activation of dorsal raphe serotonergic neurons increases pupil size. Curr Biol. 2021;31(1):192-197.e4. doi: 10.1016/j.cub.2020.09.090 33186549 PMC7808753

[78] Grujic N, Tesmer A, Bracey E, Peleg-Raibstein D, Burdakov D. Control and coding of pupil size by hypothalamic orexin neurons. Nat Neurosci. 2023;26(7):1160–4. doi: 10.1038/s41593-023-01365-w 37336973 PMC10322704

[79] Qiu Y, Park S, Oszczapinska U, Heller LM. Visual disgust constricts pupils in response to misophonic movies. Front Psychol. 2025;16:1569598. doi: 10.3389/fpsyg.2025.1569598 40873517 PMC12379095

[80] Liljequist D, Elfving B, Skavberg Roaldsen K. Intraclass correlation - A discussion and demonstration of basic features. PLoS One. 2019;14(7):e0219854. doi: 10.1371/journal.pone.0219854 31329615 PMC6645485

