# Supplementary material for: Sound-evoked pupil dilation quantifies misophonic symptoms
Source: PLoS One. 2026 May 20;21(5):e0348278. doi: 10.1371/journal.pone.0348278 (PMC13189328; doi:10.1371/journal.pone.0348278)
Supplement: Attachment — Submitted filename: deGeeEtAl_Rebuttal.pdf [file pone.0348278.s004.pdf]

UNIVERSITEIT VAN AMSTERDAM

Dept. of Psychology
University of Amsterdam
Nieuwe Achtergracht 129B
Amsterdam, The Netherlands
Tel (secret.): +31205256840
Email: R.Rouw@uva.nl

Amsterdam, June 11, 2025

Dear Editors of *PLOS One*,

Our initial submission (PONE-D-24-43249) was desk-rejected by Saswatika Beura because understood that we started data collected before obtaining ethical approval, and that, as such, we did not meet PLOS One's ethical requirements for human subjects research. This was however a miscommunication. Naturally, we only started data collection after we obtained ethical approval. In fact, the University of Amsterdam adheres to highest of ethical standards. The miscommunicated lied in the “starting date” of the project: we understood this to be the date we started working on this (e.g., on the ethical approval) and not the date we started data collection. We communicated this but never received a reply. We now write in the manuscript:

“The experiments were approved by the ethics committee of the Department of Psychology at the University of Amsterdam. Experiment 1 was approved on March 22nd, 2023 (FMG-2306) and ran from May 8th, 2023, until July 25th, 2023. Experiment 2 was approved on February 6th, 2025 (FMG-2306; amendment) and ran from February 20th, 2025, until May 16th, 2025.”

Apart from the miscommunication outlined above, we decided to perform an internal replication of our work. We have added this additional experiment to the current submission.

Sincerely Yours,

Dr. Jan Willem de Gee, Assistant Professor of Systems and Cognitive Neuroscience
Dr. Romke Rouw, Assistant Professor of Brain and Cognition
University of Amsterdam, Amsterdam, NLD
