# Supplementary material for: Sound-evoked pupil dilation quantifies misophonic symptoms
Source: PLoS One. 2026 May 20;21(5):e0348278. doi: 10.1371/journal.pone.0348278 (PMC13189328; doi:10.1371/journal.pone.0348278)
Supplement: Attachment — Submitted filename: deGeeEtAl_Rebuttal_auresp_2.pdf [file pone.0348278.s006.pdf]

We are delighted with the positive evaluation of our manuscript and thank both reviewers again for their thoughtful and constructive feedback. Below, we provide detailed responses to the reviewer's remaining comments.

REVIEWER 1

I have reviewed a previous version of this manuscript and appreciate the substantial effort in this revision, particularly in clarifying the methods and demonstrating the robustness of the findings. Most of my prior concerns have been adequately addressed, though a few points warrant further clarification to strengthen the paper prior to publication.

Thank you.

My primary remaining question concerns the pupil size contrast between misophonic triggers and generally aversive sounds. I now better understand the focus on individual differences in these contrasts rather than the group-level effect per se. That said, it was surprising to observe such robust correlations between the difference scores, which are typically less reliable than raw scores, and the rating scores. Could the authors provide reliability estimates for the pupil contrast (e.g., split-half reliability)?

Thank you for this great suggestion. We have added test-retest reliability analyses, which confirm all the reviewer's predictions (lines 302-307 and new **S2A Fig**):

> "As a first step, we assessed test–retest reliability of pupil responses separately for triggers and generally unpleasant sounds, and for the contrast between them. Reliability was high for trigger sounds ($r = 0.805$) and generally unpleasant sounds ($r = 0.758$) but lower for the contrast ($r = 0.570$) (**S2A Fig**). This reduction in reliability is consistent with the well-known tendency for difference scores to accumulate measurement error from both component conditions."

The modest test-retest reliability of the pupil contrast (median of bootstrapped distribution, $r = 0.570$) is likely a consequence of the relatively low number of sound presentations per participant (in total 40 trigger sounds and 40 generally aversive sounds). Against this background it is even more important that we have provided an internal replication of our main finding (**S2B,C Fig**).

More importantly, however, I think that discussing potential reasons for the observed pupil differences would enrich the manuscript. Although the authors note the lack of directional hypotheses, a non-zero contrast was presumably anticipated to enable correlational analyses. The authors' prior response indicated that the contrast's direction might vary with sample composition and misophonia severity; elaborating on expected patterns (in either direction), their potential replicability, and their implications for sound processing could be valuable. For instance, misophonic triggers may have received more attention, due to some of the differences from generally aversive sounds the authors mentioned. I would welcome the authors' perspective on these possibilities, given my limited expertise in this domain.

At the group level, trigger sounds elicited a larger pupil response than aversive sounds. As discussed previously, however, our primary focus is on the inter-individual variation in the pupil response contrast between these sound categories. Because we did not formulate an a priori hypothesis regarding the overall increase in sound-evoked pupil dilation, nor investigate any of the processes that potentially underlie this increased pupil response, we believe an extensive discussion of this effect to be beyond the scope of the present manuscript.

To clarify this point further, several considerations are relevant:

1. As far as we know, ours is one of only two studies examining pupil dilation in misophonia [1], limiting the opportunity for a literature review on this specific topic.
2. In contrast, the body of literature on both the determinants of increased pupil dilation, and on the potential reasons for increased misophonic responses is large and very heterogeneous. For example, attentional, social, emotional, and cognitive processes have all been brought forward as essential contributors to increased misophonic responses [2–6]. Because we neither directly measured nor specifically targeted any of these processes, we see no principled basis for selectively reviewing a subset of these factors while excluding others. We feel such discussion is thus beyond the scope of the current manuscript.
3. Most importantly, the manuscript was extensively restructured in response to concerns raised during the previous review round, specifically to avoid the misinterpretation that an effect of overall increased pupil response to misophonic sounds constitutes the main finding. We fear that including a discussion on the reasons for the observed increased pupil response to misophonic sounds would reverse that improvement. This would potentially harm the obtained clarification of the manuscript, and compromise its narrative and conceiseness.

Still, we agree with the reviewer that these are important questions, and that improved understanding of the role of attention in shaping the misophonic response is a critical question that warrants furher exploration. Accordingly, we added the following lines to the Discussion (lines 477-480):

> “Similarly, the current study did not examine which cognitive, social, or emotional factors are associated with the increased pupil response to misophonic sounds (e.g., disgust [1,7]). Exploring the role of such factors with pupillometry represent important directions for future misophonia research.”

Minor points

Thank you for clarifying the rating scale definitions, which now appear more ordinal than previously described. I remain interested in the rationale for the response input mode (three adjacent keys with a physically offset key for the highest rating). Additionally, while pupillometry offers advantages over categorical self-reports for assessing misophonia symptom severity, could a visual analogue scale achieve comparable precision (e.g., in computerized diagnostic settings)?

Thank you for your question. As discussed previously, we used a rating method that that is well-suited for a pupillometry paradigm, as it does not involve any visual input. In behavioral research, visual analogue scales can indeed be used, and have been used, to measure the severity of misophonic responses [8,9].

With regard to the choice of response keys, this setup was based on several considerations. First, both choosing these particular G, H, J keys and spacebar, and how this positioned the keyboard in the experimental setup, supported a comfortable hand posture. This way we could instruct participants to keep their hands in the same position throughout the experiment. Second, the left-to-right ordering is consistent with the tendency in our culture to order increasing magnitude, strength, or intensity from left to right (e.g., the Snarc effect [10]). Third, responses from the ring finger to the thumb are physically more comfortable and faster, as compared to mapping responses from the little finger to the index finger. Overall, our method thus facilitates fast responses, and an intuitive and easy to remember response mapping,

thereby helping to reduce errors and minnimize noise in the recorded data. In Methods we added a line (128-132):

> "To avoid ambiguity the scale is ordered to reflect the increasing negative valence/increasing arousal characteristic to misophonia, from neutral to full misophonic response. The G-to-spacebar key arrangement was selected to support a comfortable hand posture and facilitate fast and intuitive response mapping."

Regarding participant instructions, I question whether explicitly directing participants not to blink during sound presentation would have been advisable. Since the authors' data show rather average blink rates suggesting natural behavior, such an instruction might have imposed unintended inhibitory demands, potentially confounding the task. In my opinion, it was thus appropriate not to include such an instruction.

Agreed. We have changed one sentence in the Methods (lines 217-219):

> "The relatively high number of excluded participants may be explained by us not explicitly instructing them to refrain from blinking during the sounds."

REVIEWER 2

I have carefully reviewed the revised manuscript. The authors have adequately addressed my previous comments, and I am satisfied that the scientific revisions have been implemented appropriately. At this stage, I have only one minor comment and one editorial note.

Thank you.

Minor comment

In the clean copy (Lines 324–327), the newly added description of the phenomenon is interesting. It may be related to the report in Ref. [76], which suggests that, beyond the conventionally known arousal enhancement on the order of ~3 seconds, there may also be a component of enhancement that persists for a longer duration, around ~10 seconds, in a more baseline-like manner.

Thank your for raising this interesting point. We have added the following sentence to the Results (line 334-335):

> "This result may indicate that sound stimuli evoke alertness maintenance over relatively long timescales [11]".

Editorial note

I reviewed the tracked-changes version of the manuscript; however, it appears that the change-tracking may have been generated via an automatic post-hoc document comparison. As a result, some passages that are textually identical are shown as modified (in red), and there are also instances where the layout/formatting appears disrupted. Therefore, I was not able to reliably confirm whether all fine formatting issues are fully resolved in the revised version. I strongly recommend that the authors carefully check the final clean manuscript themselves and ensure, during the proofing process, that there are no unintended formatting or layout errors.

The tracked-changes version was indeed generated via an automatic post-hoc comparison. We have carefully checked all formatting and/or layout.

References:

1. Oszczapinska U, Park S, Qiu Y, Nance B, Julien M, Heller LM. The Impact of Disgusting Sounds on Pupil Diameter of Misophonic and Non-Misophonic Listeners. Psychophysiology. 2025;62: e70014. doi:10.1111/psyp.70014

2. Ash PA, Benzaquén E, Gander PE, Berger JI, Kumar S. Mimicry in misophonia: A large-scale survey of prevalence and relationship with trigger sounds. J Clin Psychol. 2024;80: 186–197. doi:10.1002/jclp.23605

3. Berger JI, Gander PE, Kumar S. A social cognition perspective on misophonia. Philos Trans R Soc Lond B Biol Sci. 2024;379: 20230257. doi:10.1098/rstb.2023.0257

4. Daniels EC, Rodriguez A, Zabelina DL. Severity of misophonia symptoms is associated with worse cognitive control when exposed to misophonia trigger sounds. PLOS ONE. 2020;15: e0227118. doi:10.1371/journal.pone.0227118

5. Savard M-A, Sares AG, Coffey EBJ, Deroche MLD. Specificity of Affective Responses in Misophonia Depends on Trigger Identification. Front Neurosci. 2022;16. doi:10.3389/fnins.2022.879583

6. Swedo SE, Baguley DM, Denys D, Dixon LJ, Erfanian M, Fioretti A, et al. Consensus Definition of Misophonia: A Delphi Study. Front Neurosci. 2022;16. doi:10.3389/fnins.2022.841816

7. Qiu Y, Park S, Oszczapinska U, Heller LM. Visual disgust constricts pupils in response to misophonic movies. Front Psychol. 2025;16: 1569598. doi:10.3389/fpsyg.2025.1569598

8. Enzler F, Loriot C, Fournier P, Noreña AJ. A psychoacoustic test for misophonia assessment. Sci Rep. 2021;11: 11044. doi:10.1038/s41598-021-90355-8

9. Kazazis S, Korsmit I, McAdams S. Triggering Misophonia: The Importance of Spectral Information, Temporal Information, and Action Identification. Auditory Perception & Cognition. 2024;7: 67–79. doi:10.1080/25742442.2024.2302295

10. Dehaene S, Bossini S, Giraux P. The mental representation of parity and number magnitude. Journal of Experimental Psychology: General. 1993;122: 371–396. doi:10.1037/0096-3445.122.3.371

11. Yamashita J, Terashima H, Yoneya M, Maruya K, Oishi H, Kumada T. Pupil Trend Reflects Suboptimal Alertness Maintenance over 10 s in Vigilance and Working Memory Performance: An Exploratory Study. eNeuro. 2024;11. doi:10.1523/ENEURO.0250-24.2024
