# Supplementary material for: Sound-evoked pupil dilation quantifies misophonic symptoms
Source: PLoS One. 2026 May 20;21(5):e0348278. doi: 10.1371/journal.pone.0348278 (PMC13189328; doi:10.1371/journal.pone.0348278)
Supplement: Attachment — Submitted filename: deGeeEtAl_Rebuttal.docx [file pone.0348278.s005.docx]

We sincerely thank the reviewers for their thoughtful and constructive feedback.

We noticed that both reviewers had several concerns about our group-level analyses, presented in original Fig 2. This made us realize that the focus of the paper was not sufficiently clear. Our key finding is the across-subjects correlation between individual misophonia severity (measured with the “Amsterdam Misophonia Scale”) and individual pupil contrast magnitude (difference between the average evoked pupil response to misophonia trigger vs generally aversive sounds), presented in original Fig 3. The group-level analyses were intended only as a prelude to this main finding. In this context, their direction and significance depend heavily on the average misophonia severity in the sample and are therefore not maximally informative as a standalone result.

To better highlight the focus of this paper, we have now introduced two separate Results sections and reordered the figures accordingly. In the first section we show that sound-evoked pupil dilation reliably predicts individual misophonia severity (**Fig 2**), and in the second section we show that trigger-evoked pupil dilation and subjective aversiveness ratings each explain unique variance in misophonia severity (**Fig 3**).

We believe these changes have substantially strengthened the study. Below, we provide detailed responses to each of the reviewers’ comments.

**REVIEWER 1**

The study presented here investigates the potential of pupillometry as an objective physiological marker for misophonia, both in terms of distinguishing it from general sound unpleasantness and in predicting the severity of misophonic symptoms within individuals. The authors define misophonia as a condition characterized by intense negative emotional, cognitive, or physiological responses to common, human-made sounds such as chewing or throat clearing. In two experiments, participants listened to sounds (presumably presented in randomized order) categorized by the authors (a priori) as either “generally unpleasant” or “misophonia triggers”, while their pupil size was continuously recorded using high-temporal-resolution eye-tracking. After each sound, participants rated its unpleasantness on a four-point categorical scale with the options “neutral” (1), “mild dislike” (2), “disgust or anger” (3), and “full misophonic reaction” (4). The authors report the following key findings: First, misophonic trigger sounds were rated as more unpleasant than generally unpleasant sounds. Second, pupil dilation from sound onset was greater for misophonic trigger sounds than for generally unpleasant sounds, even when matched on subjective aversiveness ratings. Finally, individual differences in both pupil dilation and rating contrasts predicted participants’ scores on the Amsterdam Misophonia Scale (A-MISO-S), suggesting that both measures may have diagnostic utility for assessing symptom severity.

Thank you for this accurate summary.

My overall impression of the manuscript is mixed. The topic is timely and relevant, and the manuscript is generally well written and clearly structured. The statistical analyses appear rigorous, and the visualizations are informative and well-designed. However, there are several conceptual and methodological issues that should be addressed before I can recommend the manuscript for publication. My main concerns pertain to the theoretical justification for the pupillometry approach and the key comparison, the need for clarification of specific methodological procedures, and questions regarding the interpretation of the physiological data.

Thank you for your thoughtful input, which helped us to improve the manuscript.

**Major points**

*Theoretical motivation.*

1. The authors motivate their work by emphasizing the need for objective indicators of misophonia due to its high prevalence and clinical relevance. However, the study would benefit from a stronger theoretical justification for using pupil dilation (rather than alternative physiological measures) as an indicator of the condition,

Thank you for raising this important point. We agree that the theoretical justification did not sufficiently come across. In the revised Introduction, we now spell out our rationale in more detail. Specifically, we present (i) the hypothesized circuit underlying misophonic responses (**lines 70-75**), (ii) that pupil size fluctuations at constant luminance reflect the activity of this circuit (**lines 75-77**), (iii) that pupil responses scale with the emotional intensity of auditory stimuli (**lines 77-78**), and finally (iv) the downsides of other physiological measures (**lines 78-85**).

and for the specific comparison between misophonic and generally unpleasant sounds. Pupil dilation is commonly interpreted as a marker of arousal or cognitive effort, but these are broad and relatively unspecific constructs. As the authors themselves acknowledge, pupil responses can be driven by a wide range of processes, including positive emotional reactions, not just negative responses such as those associated with misophonia.

Thank you. Please also see our explanation all the way on top of this document. Our starting point was the above theoretical foundation, including that pupil responses scale with the emotional intensity of auditory stimuli [1–7]. This predicts more pupil dilation to common trigger sounds in people with more severe misophonia. Please note that the pupil contrast (trigger vs generally aversive sounds) is crucial, as it controls for potential individual differences in hearing ability and general sound sensitivity. This contrast is a standard method in misophonia research, as it specifically reflects the effect of the misophonic trigger, rather than more general emotional, auditory or physiological (pupil) effects. We now specify this explicitly in the Materials and Methods (**lines 158-170**) and Results (**lines 295-297**) and cite earlier work using the same approach [8–13]. Additionally, in a new Discussion paragraph, we now reflect on the disadvantages of using pupillometry in the context of misophonia (**lines 450-464**).

2. Furthermore, it was unclear from the introduction whether the authors expected both higher aversiveness ratings and greater pupil dilation for misophonic trigger sounds compared to generally unpleasant sounds, as the directional hypotheses were not explicitly stated.

Thank you. Please also see our explanation all the way on top of this document. We did not have a specific hypothesis about the direction of the group-level results, as this would depend on the average misophonia severity in the study sample. We have clearly specified our main hypothesis regarding individual differences at the end of the Introduction (**lines 86-89**).

Similarly, it is not explained why pupil responses should scale with the severity of misophonic symptoms. The manuscript would benefit from a clearer theoretical framework outlining what pupil size is thought to index in this context, and how the mechanisms underlying misophonic versus generally aversive sound processing differ. Integrating these theoretical perspectives more directly into the introduction could provide a stronger rationale for the current research and the critical comparisons.?

Thank you. See our reply to point 1. Additionally, we have added a paragraph in the Materials and Methods, in which we describe the differential mechanisms underlying misophonic versus generally aversive sound processing (**lines 158-170**).

*Methodology.*

3. Regarding methodology and the interpretation of the pupil data, my first concern relates to the control of low-level stimulus properties. From my understanding (though I may be mistaken) the sounds differed within and across categories in terms of duration (10–20 seconds), loudness (30–70 dB), and likely in additional acoustic features such as onset regularity or sharpness (based on the spectrograms provided). How can it be ruled out that differences in aversiveness ratings and pupil dilation were at least partly driven by systematic differences in these low-level features? Did the authors take any steps to control such confounds? In addition to the visual representations provided, I would strongly encourage the authors to make the sound stimuli and data available (at least to reviewers) to allow for better evaluation.

Thank your raising this important point and the excellent suggestion to make all sound stimuli available. Please also see our explanation all the way on top of this document. Our main conclusion – that misophonia severity can reliably, objectively and cost-effectively be indexed by pupil-linked arousal – is afforded by the individual differences analysis (**Fig 3C**). Critically, since all participants were exposed to the exact same sounds, this analysis does not suffer from potential systematic differences in low-level acoustic features. Thus, any across-subjects variation in the magnitude of the presented pupil contrast (trigger vs generally unpleasant) must be due to intrinsic factors (plus some random measurement noise).

We agree that we cannot fully rule out that the group-level effect of sound category (trigger vs generally unpleasant) on pupil dilation (**Fig 2A,B**) or subjective rating (**Fig 3B**) may be (partly) due to differences in acoustic features. Matching for all acoustic features is however impossible, as this would distort sounds to the extent that they are no longer recognizable, and no longer members of the “misophonic trigger” sound category. For example, misophonia triggers are often soft human-made sounds (sniffing, swallowing and whispering), and loudness is an integral part of the sound stimulus. Therefore, we have opted for using the same sounds as in previous work [8,14], and, for transparency, plotting the associated spectrograms (**S1A Fig**) and making the sound stimuli publicly available (<https://doi.org/10.5281/zenodo.18202830>). We have added this text to the Materials and Methods (**lines 151-157**).

We have additionally expanded the Discussion paragraph describing the study limitations to address these points (**lines 471-477**).

4. I also have some concerns about the subjective aversiveness ratings. The four response options appear to be more qualitative than ordinal, which complicates the interpretation of mean scores (e.g., as shown in Figure 2A). Why did the authors not opt for a more conventional Likert-type scale ranging from, for instance, 1 to 5, with clearly ordered categories for aversiveness or valence, or a continuous visual analogue scale? Furthermore, it is unclear why the lowest anchor was “neutral”. Is it possible that some participants found individual sounds pleasant but had no way to indicate this due to the restricted response range?

The subjective rating scale we used is ordinal. The definition of each rating was as follows:

1. “This sound doesn’t affect me at all, the sound may be mildly annoying/disturbing.”
2. “This sound is annoying, I don’t like the sound.”
3. “I do not like this sound at all, it makes me feel very uncomfortable.”
4. “I feel angry, I feel anxiety, I feel fear, I need to flee, I want to remove the headphones, this sound makes me physically upset.”

We agree that some participants may have experienced certain sounds as pleasant, but, given the above definitions, we are confident that in those cases participants would have used the lowest (neutral) rating. The choice of four answer options from “neutral” to full “misophonic response” is to ensure unambiguous ratings. The misophonic response is defined by negative valence in combination with high-arousal activation of the sympathetic nervous system [8,15–17]. However, there is no clear emotional “opposite” of a misophonic response: is it “pleasant” (positive valence) or “serene” (positive valence, low arousal) or perhaps “depressed” or “bored” (negative valence, low arousal)? We felt that introducing an additional emotional label would confuse the underlying dimension of the scale and might elicit heterogeneous interpretations across participants. We have added a short explanation along these lines in the Materials and Methods (**lines 135-137**).

5. Participants were reportedly instructed on how to use the scale, including what constitutes a “full misophonic reaction”. This definition should be included in the manuscript for readers unfamiliar with the concept of misophonia.

Fully agreed. See our reply to point 4. We have included the definition in the Materials and Methods (**lines 129-135**).

More critically, the explicit inclusion of a “full misophonic reaction” category, especially since it was mapped to a distinct response key, may have introduced demand characteristics or bias. Could this have turned the task into something akin to a “misophonic trigger detection” task, potentially influencing both behavioral and physiological responses? For instance, did participants potentially expend more cognitive effort to determine whether a sound warranted the “full misophonic reaction” label?

Thank you. We cannot fully rule this out, but we think this is unlikely for three reasons. First, the rating definitions (see our reply to point 4) imply an ordinal scale with interval properties, Participants received the instruction to “rate their physical response” explicitly mentioning ordinality (“on a scale of 1-4"). Second, participant behavior is line with reflecting strength of response rather than whether it categorically fits with misophonia: e.g., certain trigger sounds received a rating 1 while non-trigger sounds could still receive rating 4 (**Fig 3A**). Third, all sounds had a duration of at least of 10 s, pupil dilation was measured in the first 3 s, and subjective ratings only came afterwards.

Additionally, given the instructions regarding the construct and if the generally unpleasant sounds did not contain any misophonia triggers, one would not expect any generally unpleasant sounds to receive a rating of 4. Was this the case? Given the categorical and arguably non-metric nature of the scale, I would also strongly encourage the authors to show the full distribution of ratings by sound category rather than presenting mean values, which may be inappropriate for the level of measurement.

Example generally aversive sounds are “nails on chalkboard” or “baby crying”. Occasionally participants rated these with a 4 (“I feel angry, I feel anxiety, I feel fear, I need to flee, I want to remove the headphones, this sound makes me physically upset.”), which is not unexpected. Thank you for the excellent suggestion to show full distributions of ratings by sound category; we now do so (**Fig 3A**).

**REVIEWER 1 - Minor points**

1. Section 2: The study appears to be embedded within a larger research program, given the variety of manipulations, protocols, and measures mentioned but not analyzed in the current paper. While this is not problematic per se, I would expect a preregistration or study plan indicating that the current subset of analyses and measures was part of the original plan. Without such documentation, the selection of outcomes may appear post hoc. To be clear, I do not intend to imply selective reporting, indeed, the authors are very transparent about their design choices and analyses, but it may be helpful to clarify this point earlier in the manuscript. I would prefer the full data from all protocols and conditions to be made available to the reviewers before publication.

Thank you for raising this point. Experiment 1 involved a thesis project of two MSc students: one would focus on misophonia and the other on musical frisson. Because of time constraints imposed by the MSc program, we did not have time to preregister the project, which we agree would have been better. However, please note that with Experiment 2 we provide an internal replication of our misophonia results in an independent sample (**S2A,B Fig**). There is no question of selective reporting, as we present all acquired data except those on musical frisson (Materials and Methods), which is a different topic. Importantly, frisson data were collected after misophonia sound blocks. Finally, please note that: (i) our main hypothesis brought misophonia and pupillometry literature together in a straightforward fashion (see our reply to point 1), (ii) the followed procedures, including those to measure misophonic severity, are identical to those previously followed in our lab (and were at the time already described and online available [18]) and (iii) the contrast between trigger and generally aversive sounds is a standard procedure in misophonia research [8–13].

2. Section 2.2.1 / Figure 1: The study used 10 different “generally unpleasant” and 10 different “misophonic trigger” sounds. From the description of the misophonia protocol, I gather that the two misophonia blocks were separated by a breathwork/distraction intervention in Experiment 1.

Correct. We now clearly show this (**Fig 1A**).

Figure 1D suggests that the sounds were randomly intermixed within blocks, with 20 sounds per block. Did each sound appear once per block, or twice? This could be clarified in the protocol description, and the figure could indicate block structure more clearly.

Indeed, in one block, participants were exposed twice to ten unique generally unpleasant sounds, and ten unique common misophonia trigger sounds; the resulting forty sounds were presented in fully randomized order. We have now expanded the protocol description (**lines 142-145**).

3. Section 2.3: Were participants given fixation instructions? Since pupil size is sensitive to gaze direction, it would be important to report whether participants were instructed to maintain central fixation and whether gaze position was monitored during the trials.

Participants were instructed to maintain central fixation (on a square during sound presentation, and on a diamond during their subjective rating) and we monitored gaze data. We have now added this (**lines 124-125**). Your comment inspired us to analyze saccade rate in our misophonia protocol. There was no group-level effect of sound category on saccade rate during the first three seconds of trigger sounds (same interval used to quantify pupil responses) (**S2G Fig**), and saccade rate did not predict misophonia severity (**S2H Fig**).

4. Section 2.5.2: It was unclear why trials with blinks during the first three seconds were excluded if pupil size during blinks was interpolated. Could the authors clarify this criterion?

Pupil responses time locked to blinks are large and can last for up to 8 seconds [19]: the opening of the eye after a blink causes a light transient that triggers the pupil light reflex. Therefore, the pupil size timeseries in the couple of seconds after blinks contain a lot of noise, and the conservative approach is to not consider these trials. Note that blink interpolation is required as part of the preprocessing pipeline, because subsequent temporal filtering operates on the complete time series. Our main results are qualitatively the same when we do not exclude trials with eye blinks during the first three seconds of sound presentation (**S2C,D Fig**).

5. Section 2.7: The authors used linear mixed-effects models to predict pupil dilation based on sound category and average aversiveness ratings. In addition to the general concerns raised earlier about the rating scale, I am also wondering about potential collinearity between predictors, given that ratings and sound categories were likely correlated. Was this considered or tested?

In this regression model the average (± S.E.M.; across participants) variance inflation factor (VIF) is 1.27 (± 0.06), which is considerably lower than common cutoffs 5 or 10. We have added this to the Results (**lines 369-371**).

**REVIEWER 2**

The authors investigate misophonia, a disorder where certain sounds evoke intense negative arousal responses. The study employs pupillometry to objectively measure these responses, which show distinctive pupil dilation patterns. This method could differentiate misophonic responses from other unpleasant sounds and reflect the severity of symptoms in individuals.

This manuscript presents an interesting and novel approach to objectively assess misophonia through pupillometry. However, several key concerns and clarifications are needed before the manuscript can be considered for publication. While the study design and methodology are promising, the authors should refine their explanations and analyses to ensure that the results are fully interpretable and robust. Also, the manuscript would benefit from a more thorough discussion of the limitations and potential sources of bias in the data.

Thank you for your thoughtful input, which helped us to improve the manuscript.

**Major points**

1. Data Preprocessing: The extensive preprocessing applied to the pupil diameter data raises some concerns. While rigorous noise removal is important, overly complex preprocessing could reduce the transparency and reproducibility of the analysis. Would similar results be obtained with a simpler preprocessing approach, such as blink removal and linear interpolation, applied to the pupil data?

Thank you for raising this concern. We have repeated the main analysis after only linearly interpolating blinks and converting the block-wise time series to units of percent signal change. Our main results are qualitatively the same (**S2E,F Fig**). We have added this to the Results text (**lines 321-324**).

2. Potential Confounding Factors: The study could benefit from addressing the potential impact of time-scale effects on pupil dilation. One potentially uncontrolled factor is the time-scale effects on pupil diameter. It is known that arousal fluctuations influencing task performance can change with rhythms in the 10-16 second range (<https://doi.org/10.1523/ENEURO.0250-24.2024>), and the stimuli in this study were presented in cycles that align with this period. Additionally, reports suggest that pupil diameter tends to decrease over the course of a long experiment (<https://doi.org/10.1371/journal.pone.0165274>), as also seen in Figure 1D. These points should at least be mentioned as a limitation in the study.

We agree that time-scale effects on pupil dilation is a potential limitation of the study. Indeed, pupil responses tend to be larger early in the block.

**Figure R1.** Average sound-evoked pupil response separately per sound category (orange, trigger; blue, generally aversive) and trial number.

However, since we fully randomized sounds within blocks, time-scale effects can only add noise and not drive the result. We have expanded the Discussion paragraph describing the study limitations to address these points (**lines 461-464**).

3. Possible Setup issue?: The exclusion of about 30 participants due to blinks is notable. While it is good that the authors have shown that the results remain the same without these exclusions, why were such a large number of blink intervals recorded for most participants? It seems unusual for participants to blink this often everyday activities. Is there something about the (unusual) experiment setup that could be contributing to this?

We excluded trials in which participants blinked during the first three seconds of sound presentation (Materials and Methods) and excluded participants for which more than two-thirds of trials had to be excluded due to blinking (N=30, grey data points in **Fig 1B** and **S1B,C Fig**; Materials and Methods). The relatively high number of excluded participants can be explained by our failure to explicitly instruct them not to blink during the sounds. The average blink rate during the first three seconds of sound presentation was 19.5 blinks per minute (**S1C Fig**), which is close to the average human spontaneous blink rate of ~17 blinks per minute [20,21].

We have expanded the Materials and Methods section on exclusion criteria to reflect these points (**lines 224-228**).

4. Stimulus Variability: The current analysis primarily focuses on individual differences. It would be beneficial to also examine intra-individual variability in response to different stimuli. The authors mention that the sample size is too small to examine differences between stimuli, but can any statistical measures of variability be computed between stimuli? Even without a full stimulus-by-stimulus analysis, could provide valuable insights into misophonic responses.

Thank you for this nice suggestion. One possibility is that pupil response variability is smaller for trigger vs generally unpleasant sounds, and it could be that this is especially true for those individuals with severe misophonia. However, we did not find evidence for this: at the group-level there was no difference in pupil response variability between triggers and generally aversive sounds (**S2I Fig**); likewise, there was no significant relationship between A-MISO-S scores and individual pupil response variability contrast (**S2J Fig**). We have added this to the Results (**lines 328-333**).

5. Rating Methodology: It seems that four distinct qualitative categories were converted into a quantitative scale (1-4). The authors should justify this methodology or consider alternative ways of analyzing subjective ratings of stimuli.

Thank you. See our reply to reviewer 1 major point 5.

6. Time Window for Pupil Dilation Measurement: The rationale for the specific 1-3 second window following stimulus presentation should be explained in more detail. A clearer justification would help readers understand the significance of this timing.

Thank you. Our time window was based on the known pupil impulse response function [22] and the same as in recent pupillometry during sound presentation [23,24]. Your comment inspired us to (exploratively) try a much broader window of interest. Interestingly, using the time window 0 – 10 seconds from sound onset made our main result even stronger:

We have expanded the Materials and Methods section on analysis of eye data (**lines 250-254**) and the Results (**lines 324-327**) to reflect these points.

7. Operational Definitions: A key issue I had concerns the operational definition of what distinguishes “unpleasant” sounds from “trigger” sounds. The operational definitions of these categories should be well-defined and justified to avoid ambiguity in the results. If the A-MISO-S score is treated as a reference for "true" misophonic severity, is this assumption valid? Is the difference between "trigger" and "unpleasant" sounds based on a specific, clear criterion, and how does this relate to the results?

Thank you for this important question. Please see our replies to reviewer 1 major points 1 and 2. We now provide an operational definition of what distinguishes triggers from generally unpleasant sounds (**lines 158-170**). In short, generally unpleasant sounds refer to a selection of sounds whose specific acoustic properties evoke negative emotional responses (these acoustic properties are not shared with misophonic trigger sounds). The negative arousal responses make unpleasant sounds a more stringent control condition than neutral sounds, while the distinct characterization of misophonia (see our reply to reviewer 1 major point 1) entails that the contrast of trigger versus unpleasant sounds reflects the specifically more intense response to misophonic trigger sounds than to these control stimuli.

Concerning the A-MISO-S, the questionnaire score is a reference for true misophonic severity. While misophonia remains a topic of research, there is evidence that the current understanding and characterization of the condition can be reliably captured with the A-MISO-S questionnaire [25–27] and that the scale is sensitive to variations in the severity of misophonic complaints, such as misophonic symptom reduction following treatment [28–30]. We have added this to the Materials and Methods (**lines 201-205**).

8. Sound-evoked Pupil Responses: Even if there were clear, operational criteria for distinguishing unpleasant from trigger, it remains questionable whether the contrast between unpleasant and trigger itself produced this difference. It is unclear whether other factors, such as loudness, subjective saliency (<https://doi.org/10.3758/s13423-015-0898-0>) or emotional responses (<https://doi.org/10.1016/j.biopsycho.2025.109044>), could be contributing to these effects. After controlling all these factors, is it valid to conclude that the contrast between unpleasant and trigger explains the observed differences in pupil dilation?

Thank you for this important question. Please see our explanation all the way on top of this document, and our reply to reviewer 1 major point 3. We now cite the mentioned papers.

9. Theoretical Considerations: Lines 323-324 suggest that the combination of a categorical scale with continuous pupil diameter data improves explanatory power. However, isn't it possible that these measures are assessing different aspects? The relationship between pupil diameter and arousal, as well as the differences between continuous and categorical measures, could be more thoroughly explored in the discussion. The theoretical basis for combining these measures should be better explained.

Agreed. See also our reply to reviewer 1 major points 4 and 5. We have expanded the Discussion paragraph describing the study limitations to address these points (**lines 480-484**).

**Minor points**

Lines 251-252: Lines 251-252 are unclear. Could the authors clarify what sample is referred to as 8 and what the sentence is meant to convey?

Done

**References**

1.	Joshi S, Gold JI. Context-dependent relationships between locus coeruleus firing patterns and coordinated neural activity in the anterior cingulate cortex. Elife. 2022;11: e63490. doi:10.7554/eLife.63490

2.	de Gee JW, Colizoli O, Kloosterman NA, Knapen T, Nieuwenhuis S, Donner TH. Dynamic modulation of decision biases by brainstem arousal systems. eLife. 2017;6: 309.

3.	Murphy PR, O’Connell RG, O’Sullivan M, Robertson IH, Balsters JH. Pupil diameter covaries with BOLD activity in human locus coeruleus. Human brain mapping. 2014;35: 4140–4154.

4.	Lloyd B, de Voogd LD, Mäki-Marttunen V, Nieuwenhuis S. Pupil size reflects activation of subcortical ascending arousal system nuclei during rest. Elife. 2023;12: e84822. doi:10.7554/eLife.84822

5.	Bradley MM, Miccoli L, Escrig MA, Lang PJ. The pupil as a measure of emotional arousal and autonomic activation. Psychophysiology. 2008;45: 602–607. doi:10.1111/j.1469-8986.2008.00654.x

6.	Joshi S, Gold JI. Pupil Size as a Window on Neural Substrates of Cognition. Trends in Cognitive Sciences. 2020 [cited 22 Apr 2020]. doi:10.1016/j.tics.2020.03.005

7.	Joshi S, Li Y, Kalwani RM, Gold JI. Relationships between Pupil Diameter and Neuronal Activity in the Locus Coeruleus, Colliculi, and Cingulate Cortex. Neuron. 2016;89: 221–234.

8.	Edelstein M, Brang D, Rouw R, Ramachandran VS. Misophonia: physiological investigations and case descriptions. Front Hum Neurosci. 2013;7. doi:10.3389/fnhum.2013.00296

9.	Kumar S, Tansley-Hancock O, Sedley W, Winston JS, Callaghan MF, Allen M, et al. The Brain Basis for Misophonia. Curr Biol. 2017;27: 527–533. doi:10.1016/j.cub.2016.12.048

10.	Siepsiak M, Rosenthal MZ, Raj-Koziak D, Dragan W. Psychiatric and audiologic features of misophonia: Use of a clinical control group with auditory over-responsivity. J Psychosom Res. 2022;156: 110777. doi:10.1016/j.jpsychores.2022.110777

11.	Kumar S, Dheerendra P, Erfanian M, Benzaquén E, Sedley W, Gander PE, et al. The Motor Basis for Misophonia. J Neurosci. 2021;41: 5762–5770. doi:10.1523/JNEUROSCI.0261-21.2021

12.	Savard M-A, Sares AG, Coffey EBJ, Deroche MLD. Specificity of Affective Responses in Misophonia Depends on Trigger Identification. Front Neurosci. 2022;16. doi:10.3389/fnins.2022.879583

13.	Daniels EC, Rodriguez A, Zabelina DL. Severity of misophonia symptoms is associated with worse cognitive control when exposed to misophonia trigger sounds. PLOS ONE. 2020;15: e0227118. doi:10.1371/journal.pone.0227118

14.	Kumar S, Forster HM, Bailey P, Griffiths TD. Mapping unpleasantness of sounds to their auditory representation. J Acoust Soc Am. 2008;124: 3810–3817. doi:10.1121/1.3006380

15.	Swedo SE, Baguley DM, Denys D, Dixon LJ, Erfanian M, Fioretti A, et al. Consensus Definition of Misophonia: A Delphi Study. Front Neurosci. 2022;16. doi:10.3389/fnins.2022.841816

16.	Grossini E, Stecco A, Gramaglia C, De Zanet D, Cantello R, Gori B, et al. Misophonia: Analysis of the neuroanatomic patterns at the basis of psychiatric symptoms and changes of the orthosympathetic/ parasympathetic balance. Front Neurosci. 2022;16: 827998. doi:10.3389/fnins.2022.827998

17.	Jastreboff PJ, Jastreboff MM. The neurophysiological approach to misophonia: Theory and treatment. Front Neurosci. 2023;17. doi:10.3389/fnins.2023.895574

18.	Cerliani L, Rouw R. Increased orbitofrontal connectivity in misophonia. bioRxiv; 2020. p. 2020.10.29.346650. doi:10.1101/2020.10.29.346650

19.	Knapen T, de Gee JW, Brascamp J, Nuiten S, Hoppenbrouwers S, Theeuwes J. Cognitive and Ocular Factors Jointly Determine Pupil Responses under Equiluminance. PLOS ONE. 2016;11: e0155574.

20.	Tomashin A, Fusina F, Marino M, Angrilli A. The eye, a spy hole on human mind: Spontaneous blink rate and amplitude, and their variability, as new psychobiological markers of anxiety. PLoS One. 2025;20: e0338262. doi:10.1371/journal.pone.0338262

21.	Bentivoglio AR, Bressman SB, Cassetta E, Carretta D, Tonali P, Albanese A. Analysis of blink rate patterns in normal subjects. Mov Disord. 1997;12: 1028–1034. doi:10.1002/mds.870120629

22.	Hoeks B, Levelt WJM. Pupillary dilation as a measure of attention: a quantitative system analysis. Behavior Research Methods, Instruments, & Computers. 1993;25: 16–26.

23.	Becker J, Viertler M, Korn CW, Blank H. The pupil dilation response as an indicator of visual cue uncertainty and auditory outcome surprise. Eur J Neurosci. 2024;59: 2686–2701. doi:10.1111/ejn.16306

24.	Doll L, Heiland S, Gutschalk A. A Role of Pupil-linked Arousal, Cingulo-insular Cortex, and Intralaminar Thalamus for Auditory Near-threshold Perception. J Cogn Neurosci. 2025;37: 1391–1415. doi:10.1162/jocn_a_02324

25.	Naylor J, Caimino C, Scutt P, Hoare DJ, Baguley DM. The Prevalence and Severity of Misophonia in a UK Undergraduate Medical Student Population and Validation of the Amsterdam Misophonia Scale. Psychiatr Q. 2021;92: 609–619. doi:10.1007/s11126-020-09825-3

26.	Schröder A, Vulink N, Denys D. Misophonia: Diagnostic Criteria for a New Psychiatric Disorder. PLOS ONE. 2013;8: e54706. doi:10.1371/journal.pone.0054706

27.	Cakiroglu S, Cosgun S, Gormez V. The prevalence and severity of misophonia in the Turkish population and validation of the Amsterdam Misophonia Scale-Revised. Bull Menninger Clin. 2022;86: 159–180. doi:10.1521/bumc.2022.86.2.159

28.	Schneider RL, Arch JJ. Case study: A novel application of mindfulness- and acceptance-based components to treat misophonia. Journal of Contextual Behavioral Science. 2017;6: 221–225. doi:10.1016/j.jcbs.2017.04.003

29.	Reid AM, Guzick AG, Gernand A, Olsen B. Intensive cognitive-behavioral therapy for comorbid misophonic and obsessive-compulsive symptoms: A systematic case study. Journal of Obsessive-Compulsive and Related Disorders. 2016;10: 1–9. doi:10.1016/j.jocrd.2016.04.009

30.	Kamody RC, Del Conte GS. Using Dialectical Behavior Therapy to Treat Misophonia in Adolescence. Prim Care Companion CNS Disord. 2017;19: 17l02105. doi:10.4088/PCC.17l02105
