# Supplementary material for: Sound-evoked pupil dilation quantifies misophonic symptoms
Source: PLoS One. 2026 May 20;21(5):e0348278. doi: 10.1371/journal.pone.0348278 (PMC13189328; doi:10.1371/journal.pone.0348278)
Supplement: Attachment — Submitted filename: deGeeEtAl_Rebuttal_.docx [file pone.0348278.s007.docx]

Below, we provide detailed responses to the reviewer’s remaining comments, which were communicated to us via email:

R1 argues that several claims in the manuscript are based on difference scores (typically low in reliability) which require addressing because the interpretability of any correlation depends on the reliability of the measures involved. R1 therefore requests that appropriate reliability estimates (e.g., split-half or test–retest) be provided, and that the implications of this reliability for the interpretation of the correlations be clarified in the discussion.

Thank you. We have added intra-class correlation coefficients [1], based on split-half, in the figure legends of S2A Fig and S3C Fig. We have also added the following sentence to the Discussion: “Second, the signal-to-noise ratio of pupil responses is limited, as illustrated by the modest test-retest reliability of the pupil contrast (**S2A Fig**; ICC = 0.497). This level of reliability implies that the observed correlation with misophonia severity is likely an underestimate of the true association, because measurement error in the predictor attenuates observed correlations. Thus, the reliability estimate qualifies the precision of the effect size while supporting, rather than undermining, the robustness of the reported relationship.”

R2 argues that because the study uses pupil size as a central measure; pupil size is influenced by general arousal, which comprises both phasic and tonic components, and both components can affect performance. Under these conditions, pupil-based effects may reflect general arousal, and this again is critical to the question of what target construct is under study, which potentially could introduces a confound in the interpretation of the findings. R2 therefore requests that this issue be addressed at a theoretical level.

We focussed on phasic (evoked) pupil-linked arousal, which was computed as the amount of pupil dilation with respect to the pre-sound pupil size. Thus accounting for any general arousal changes. This is explained in the Methods section: “We computed task-evoked pupil response measures for each trial as the mean of the pupil diameter modulation values in the window 1 s to 3 s from sound onset, minus the mean pupil size during the 0.25 s before sound onset.” This approach is established in the field [2]. We do not believe further additions are required.

The description added in lines 324–327 should be more explicitly connected to prior work suggesting a longer-lasting (~10 s) component of arousal. I would suggest considering some relevant sources (e.g., Yamashita et al., doi:10.1371/journal.pone.0276205; Welhaf, 2026, doi:10.3389/fpsyg.2026.1734451), which seem to address the mechanism that R2 is pointing to more clearly (the first focusing on pupil reactivity, the second on the underlying regulation of alertness). Of course, you may decide yourself how to integrate this. It is clear that these examples are based on performance-task settings; they do address the issue, but in a performance domain, whereas your study is situated in a perception/experience domain, so this might be worth acknowledging briefly when incorporating this line of argument.

See our reply to the previous point. We agree that characterizing the duration of arousal and alertness responses would be valuable. However, the current experimental design is not suited to address this question: immediately following each sound, participants provided a subjective aversiveness rating via button press, which itself elicits phasic arousal responses [3]. This question therefore falls outside the scope of the present paper and remains an important direction for future research.

Another point concerns the final presentation of the manuscript. I ask you to carefully check the clean version to ensure that all formatting, layout, and text integrity issues are fully resolved. In particular, please make sure that no artefacts from automated change-tracking or document comparison remain (see R2 comment for details).

We have carefully checked formatting, layout and text integrity.

**References:**

1.	Liljequist D, Elfving B, Roaldsen KS. Intraclass correlation – A discussion and demonstration of basic features. PLOS ONE. 2019;14: e0219854. doi:10.1371/journal.pone.0219854

2.	Fink L, Simola J, Tavano A, Lange E, Wallot S, Laeng B. From pre-processing to advanced dynamic modeling of pupil data. Behav Res. 2024;56: 1376–1412. doi:10.3758/s13428-023-02098-1

3.	de Gee JW, Knapen T, Donner TH. Decision-related pupil dilation reflects upcoming choice and individual bias. Proceedings of the National Academy of Sciences of the United States of America. 2014;111: E618-25.
